# Sex differences in the associations of body size and body shape with platelets in the UK Biobank cohort

**DOI:** 10.1186/s13293-023-00494-y

**Published:** 2023-02-22

**Authors:** Sofia Christakoudi, Konstantinos K. Tsilidis, Evangelos Evangelou, Elio Riboli

**Affiliations:** 1 Department of Epidemiology and Biostatistics, School of Public Health, Imperial College London, St Mary’s Campus, Norfolk Place, London, W2 1PG UK; 2 Department of Inflammation Biology, School of Immunology and Microbial Sciences, King’s College London, London, UK; 3 Department of Hygiene and Epidemiology, University of Ioannina School of Medicine, Ioannina, Greece

**Keywords:** Obesity, Body shape, Waist size, Hip size, ABSI, Platelets

## Abstract

**Background:**

Obesity is accompanied by low-grade inflammation and leucocytosis and increases the risk of venous thromboembolism. Associations with platelet count, however, are unclear, because several studies have reported positive associations only in women. Associations with body shape are also unclear, because waist and hip circumferences reflect overall body size, as well as body shape, and are correlated strongly positively with body mass index (BMI).

**Methods:**

We evaluated body shape with the allometric body shape index (ABSI) and hip index (HI), which reflect waist and hip size among individuals with the same weight and height and are uncorrelated with BMI. We examined the associations of BMI, ABSI, and HI with platelet count, mean platelet volume (MPV), and platelet distribution width (PDW) in multivariable linear regression models for 125,435 UK Biobank women and 114,760 men. We compared men with women, post-menopausal with pre-menopausal women, and older (≥ 52 years) with younger (< 52 years) men.

**Results:**

BMI was associated positively with platelet count in women, more strongly in pre-menopausal than in post-menopausal, and weakly positively in younger men but strongly inversely in older men. Associations of BMI with platelet count were shifted towards the inverse direction for daily alcohol consumption and current smoking, resulting in weaker positive associations in women and stronger inverse associations in men, compared to alcohol ≤ 3 times/month and never smoking. BMI was associated inversely with MPV and PDW in pre-menopausal women but positively in post-menopausal women and in men. ABSI was associated positively with platelet count, similarly in women and men, while HI was associated weakly inversely only in women. ABSI was associated inversely and HI positively with MPV but not with PDW and only in women. Platelet count was correlated inversely with platelet size and positively with leucocyte counts, most strongly with neutrophils.

**Conclusions:**

Competing factors determine the associations of BMI with platelet count. Factors with sexually dimorphic action (likely thrombopoietin, inflammatory cytokines, or cortisol), contribute to a positive association, more prominently in women than in men, while age-dependent factors (likely related to liver damage and fibrosis), contribute to an inverse association, more prominently in men than in women.

**Supplementary Information:**

The online version contains supplementary material available at 10.1186/s13293-023-00494-y.

## Highlights

- We examined a large middle-aged population-based cohort, excluding participants with prevalent cancer, endocrine and metabolic conditions, and severe illnesses.
- Body mass index (BMI) was associated positively with platelet count in women (strongest in pre-menopausal women) and inversely in men (strongest in older men), but inversely with platelet size in pre-menopausal women and positively in post-menopausal women and men.
- Alcohol consumption and smoking shifted the association of BMI with platelet count towards the null for women and towards a stronger inverse association for men, but did not affect the associations with waist or hip size.
- Waist size was associated positively with platelet count in women and men, with only a weak inverse association for hip size.
- Waist size was associated inversely and hip size positively with platelet size, mainly in women.

**Supplementary Information:**

The online version contains supplementary material available at 10.1186/s13293-023-00494-y.

## Background

Obesity is associated with chronic low-grade inflammation and a higher risk of venous thromboembolism [1, 2] and contributes to the development of cardiovascular diseases and some cancers [3, 4]. Although platelets have long been considered mainly participants in haemostasis and thrombosis, it is clear now that they also act as inflammatory effector cells and modulate leucocyte-mediated sterile inflammatory processes, such as atherosclerosis [5]. Platelets carry angiogenic growth factors which facilitate cancer development and, when activated by cancer cells, regulate immune cell migration towards the tumour cite and contribute to cancer metastasis and progression [5, 6]. Correspondingly, high platelet count, even when remaining within the clinical reference range, has been associated with cancer development [7, 8]. Further to platelet count, large platelet size, as reflected in large mean platelet volume (MPV), and variable platelet size, as reflected in large platelet size distribution width (PDW), are indicative of platelet activation and thrombosis [9, 10], and have also been associated with diabetes mellitus, diabetes-related complications, and some cancers [11].

We have previously shown that in UK Biobank, inflammatory biomarkers represented by C-reactive protein and leucocyte subtypes, including lymphocytes, monocytes, and neutrophils, were higher for higher body mass index (BMI), both in women and men [12]. Associations of platelet count with obesity, however, are unclear. Studies investigating platelets in relation to obesity are small scale and limited. Some have not examined separately women and men [13, 14] and those that have separated them have reported higher platelet count with obesity only in women but not in men [15, 16]. Studies on platelet size are also small scale and limited, reporting an inverse correlation of MPV with platelet count, but larger platelet size in obesity [17].

Unclear is also the association of body shape with platelets, not least because waist and hip circumferences, traditionally used to evaluate body shape, are dependent on overall body size and are correlated strongly positively with BMI [18]. The allometric body shape index (ABSI) and hip index (HI), on the other hand, reflect waist and hip size among individuals with the same weight and height and are uncorrelated with BMI [19, 20]. We have previously shown that in UK Biobank, mainly ABSI but not HI were associated with inflammatory biomarkers, represented by C-reactive protein (CRP), neutrophils and monocytes and only lymphocytes resembled glycemia-related biomarkers, with an additional inverse association with hip size [12]. Clarifying the associations of body shape with platelets matters, because the cardiometabolic complications of obesity depend on fat distribution, with visceral fat being associated with higher risk and gluteofemoral fat with lower risk [21].

In this study, we have used UK Biobank data to examine the association of body size and body shape, evaluated with allometric anthropometric indices, with platelet count and size. To clarify sex differences, we have examined separately and have compared men with women. To clarify the potential influence of endogenous sex steroids, we have compared post-menopausal with pre-menopausal women, which differ dramatically with respect to oestrogen production, and older with younger men. We have also examined heterogeneity of the associations with body size according to alcohol consumption and smoking, to account for differences in these lifestyle characteristics between women and men, and heterogeneity in the associations with body shape according to body size.

## Methods

### Study population

UK Biobank includes half a million individuals from the general population of England, Scotland, and Wales, living within 40 km of an assessment centre and aged 40–70 years at recruitment (between 2006 and 2010) [22]. As in our previous study examining associations of body size and body shape with metabolic and inflammatory biomarkers [12], we restricted the data set to participants with self-reported white ancestry and excluded participants with missing or extreme anthropometric measurements, genetically determined sex not matching the self-reported sex, and pregnant women. To minimise the influence of underlying medical conditions, which could influence body size and shape and haematological and biomarker measurements, we similarly excluded participants with prevalent cancer at recruitment (defined as in [23]), incident cancer or death within 2 years after recruitment, self-reported diabetes mellitus, or endocrine non-cancer illness, or chronic respiratory disease, or inflammatory bowel disease, or liver disease, or kidney or heart failure at recruitment, or receiving lipid lowering drugs, or exogenous steroids, or anti-hypertensive drugs at recruitment. For this study, we additionally excluded participants with cardiovascular and haematological conditions, or receiving anticoagulants, or missing all platelet measurements. The total number of excluded participants was 262,218 (52.2%). For further details on exclusions see Additional file 1: Table S1.

### Anthropometric indices

Trained UK Biobank technicians measured waist circumference at the natural indent or the umbilicus and hip circumference at the widest point [24]. We calculated ABSI for women and men and HI for women with coefficients from the National Health and Nutrition Examination Survey (NHANES) [19, 20], multiplying ABSI by 1000 to obtain numbers in the magnitude of waist circumference. For HI in men, we used coefficients previously derived from UK Biobank data [18], because HI, calculated with the original coefficients from NHANES, was correlated inversely with BMI in UK Biobank men [23]. (WC—waist circumference; HC—hip circumference)

$${\text{ABSI }} = { 1}000 \times {\text{WC}}\left( {\text{m}} \right) \times {\text{Weight}}\left( {{\text{kg}}} \right)^{{ - {2}/{3}}} \times {\text{Height}}\left( {\text{m}} \right)^{{{5}/{6}}} .$$

$${\text{HI}}_{{{\text{women}}}} = {\text{ HC}}\left( {{\text{cm}}} \right) \times {\text{Weight}}\left( {{\text{kg}}} \right)^{{ - 0.{482}}} \times {\text{Height}}\left( {{\text{cm}}} \right)^{{0.{31}0}} .$$

$${\text{HI}}_{{{\text{men}}}} = {\text{ HC}}\left( {{\text{cm}}} \right) \times {\text{Weight}}\left( {{\text{kg}}} \right)^{{ - {2}/{5}}} \times {\text{Height}}\left( {{\text{cm}}} \right)^{{{1}/{5}}} .$$

$${\text{BMI }} = {\text{ Weight}}\left( {{\text{kg}}} \right) \times {\text{Height}}\left( {\text{m}} \right)^{{ - {2}}} .$$

To standardise the anthropometric indices on a continuous scale, we calculated sex-specific *z*-scores (value minus mean, divided by standard deviation, SD). Similarly to our previous study [18], we dichotomised ABSI (cutoffs ≥ 73 for women, ≥ 80 for men) and HI (cutoffs ≥ 64 for women, ≥ 49 for men) and defined body shape phenotypes with an ABSI-by-HI cross-classification as “pear”—small-ABSI–large-HI (reference), “slim”—small-ABSI–small-HI, “wide”—large-ABSI–large-HI, “apple”—large-ABSI–small-HI. For categorisation of BMI, we used World Health Organisation criteria and defined normal weight (BMI ≥ 18.5 to < 25 kg/m^2^, reference), overweight (BMI ≥ 25 to < 30 kg/m^2^), and obese (BMI ≥ 30 to < 45 kg/m^2^). To examine heterogeneity of the associations with body shape according to body size, we defined a combined cross-classification BMI-by-ABSI-by-HI (“pear” normal weight reference).

### Haematological and biomarker measurements

UK Biobank participants provided blood samples throughout the day (8 am to 9 pm), irrespective of fasting status. Samples for haematological measurements were collected in EDTA (ethylenediaminetetraacetic acid) vacutainers and were analysed within 24 h of blood draw on Beckman Coulter LH750 automated analysers [25]. The analysers measured directly platelet count (10^9^/L), but derived platelet size, represented by MPV (fL) and PDW (%), from scatter plots and histograms of platelet size. In very few participants, haematological measurements were recorded as zero and we replaced these with half of the lowest non-zero value. We have previously described the measurements for metabolic and inflammatory biomarkers, liver function test, sex hormone binding globulin (SHBG), and sex steroids [12, 26]. In this study, we examined only the correlation of blood biomarkers with platelet count and size. As our previous study on sex steroids included fewer exclusions [26], we imputed testosterone in women using, as previously, quantile regression imputation of truncated left-censored data (QRILC) [27], but estimated the parameters of the distribution based on the restricted data set of the current study. In addition, as previously [26], we calculated free testosterone with law-of-mass-action equations [28].

To mitigate the influence of right-skewness of the distributions, we log-transformed all haematological and biomarker measurements. To provide a standardised scale for comparability, we calculated sex-specific *z*-scores.

### Statistical analysis

We examined separately women overall and men overall and the following subgroups: pre-menopausal and post-menopausal women, and younger (< 52 years at recruitment) and older (≥ 52 years) men. Menopausal status was self-reported, considering women with bilateral oophorectomy as post-menopausal and women with hysterectomy as undetermined menopausal status [12]. The age cutoff in men was selected to provide mean ages of the subgroups in men comparable to the mean ages of the subgroups by menopausal status in women.

We calculated SD differences (95% confidence intervals) for each platelet parameter with multivariable linear regression models using three combinations of the anthropometric indices as exposure variables. To examine associations with body size and body shape independent of each other, we used an additive model including BMI, ABSI, and HI on a continuous scale (interpreting estimates as SD difference in platelet parameters per one SD increment of the anthropometric index). To examine associations with body shape phenotypes, we used an additive model including body shape phenotypes (ABSI-by-HI cross-classification, “pear” reference, “slim”, “wide”, “apple”) and BMI categories (normal weight reference, overweight, obese) (interpreting estimates as SD difference in platelet parameters compared to the reference category). To examine heterogeneity of the associations with body shape according to body size, we used an interaction model including BMI-by-ABSI-by-HI cross-classification. As in our previous study [12], we adjusted all models for height (continuous), age at recruitment (continuous), weight change within the last year preceding recruitment (weight loss, stable weight, weight gain), smoking status (never, former occasional, former regular, current), alcohol consumption (≤ 3 times/month, ≤ 4 times/week, daily), physical activity (active, moderately active, inactive), Townsend deprivation index (sex-specific tertiles), region of the assessment centre, time of blood collection (8:00 to < 12:00, 12:00 to < 16:00, 16:00 to ≤ 20:15), fasting time (0–2 h, 3–4 h, ≥ 5 h), use of nonsteroidal anti-inflammatory drugs (NSAIDs), paracetamol use, and for women also menopausal status (pre-menopausal, post-menopausal, undetermined, only for women overall), HRT use (never or former, for women overall and post-menopausal), oral contraceptives use (never or former), and age at the last live birth (no live births, < 30 years, ≥ 30 years). We replaced the limited number of missing values for covariates (< 2%, see Additional file 1: Table S2) with the median sex-specific value or category.

We used Wald tests to evaluate the statistical significance of individual terms. Separately for women and men and within subgroups by menopausal status and age, we used likelihood ratio tests to evaluate the significance of the associations with body shape phenotypes overall, comparing a model including BMI categories and covariates with a model additionally including ABSI-by-HI cross-classification, as in our previous study [12]. To evaluate heterogeneity of the associations with body shape phenotypes according to BMI, we compared with a likelihood ratio test the additive model including ABSI-by-HI cross-classification, BMI categories, and covariates, with the interaction model including BMI-by-ABSI-by-HI cross-classification and covariates. To evaluate heterogeneity of the associations with anthropometric indices according to alcohol consumption or smoking status, we compared with a likelihood ratio test models with and without interaction terms of BMI, ABSI, or HI with alcohol consumption or smoking status categories (a separate model for each combination of an anthropometric index and a lifestyle variable).

To evaluate differences in the associations of body size with platelet parameters according to sex, we used an interaction term of BMI with sex (men vs. women) from a model including women and men, adjusted for ABSI, HI, and all covariates (except female-specific), and additionally including an interaction term of age with sex, to account for potential differences by menopausal status in women. To evaluate differences in the associations of body shape with platelet parameters according to sex, we used interaction terms of ABSI and HI with sex, both in the same model, including women and men and adjusted for BMI and all co-variates (except female-specific) and also including an interaction term of age with sex. To evaluate differences according to menopausal status in women, we similarly used interaction terms of anthropometric indices with menopausal status (post-menopausal vs. pre-menopausal). To evaluate differences according to age in men, we used interaction terms with age on a continuous scale. To evaluate differences in body shape phenotypes between women and men, we used likelihood ratio tests comparing fully adjusted models (including an interaction of age with sex) with and without interaction terms of ABSI-by-HI or BMI-by-ABSI-by-HI with sex. Tests for statistical significance were two-sided. We considered *p* < 0.05 as suggestive, *p* < 0.001 as evidence for association (equivalent to Bonferroni correction for 50 comparisons), and *p* < 1 × 10^–6^ as strong evidence for association (equivalent to Bonferroni correction for 50,000 comparisons).

To examine the associations of platelet parameters with liver function test, metabolic, inflammatory, and sex steroid biomarkers, we calculated partial Pearson correlation coefficients with adjustment for BMI, ABSI, and HI (continuous scale, *z*-scores), and covariates (except for region of the assessment centre), in a subset of participants with available all biomarker measurements.

In sensitivity analyses, we examined fully adjusted additive models, including BMI categories and tertiles of ABSI and HI, to explore potential non-linearity. To explore the influence of recent weight change, we restricted the data set to participants with stable weight within the year preceding recruitment. To explore the influence of medication use, we restricting the data set to participants not receiving NSAIDs or paracetamol. To explore the influence of covariates, we examined unadjusted models.

We used R version 4.1.3 for all analyses [29].

## Results

### Cohort characteristics

The study included 125,435 women and 114,760 men. Women overall had lower BMI and ABSI compared to men and lower PDW but higher platelet count and MPV (Table 1). Post-menopausal women had higher BMI and ABSI and a substantially larger proportion of “wide” phenotype compared to pre-menopausal women, but lower platelet counts and MPV. Older men also had higher ABSI and a substantially larger proportion of “wide” phenotype compared to younger men, as well as lower platelet counts and MPV, but had similar BMI (Table 1). Men were more likely to be current smokers, or to consume alcohol daily, as we have previously described [12], but in both sexes, there was no major overlap between daily alcohol consumption and current smoking (Additional file 1: Table S2). Post-menopausal women and older men were more likely to consume alcohol daily compared to pre-menopausal women and younger men, correspondingly, but were less likely to be current smokers (Additional file 1: Table S2), as we have previously described for a larger part of the UK Biobank data set [26].

**Table 1:** Anthropometric characteristics and platelet parameters of study participants

	Women			Men		
	Overall	Pre-MP	Post-MP	Overall	< 52 years	≥ 52 years
Cohort: n (% sex)	125,435 (52.2)	37,917 (30.2)	75,491 (60.2)	114,760 (47.8)	44,593 (38.9)	70,167 (61.1)
Age (years)^a,^***	55.5 (7.9)	46.6 (3.9)	60.1 (5.3)	55.0 (8.1)	46.3 (3.3)	60.4 (4.8)
Anthropometric characteristics^a,^***						
BMI (kg/m^2^)	26.2 (4.4)	25.9 (4.6)	26.2 (4.3)	27.1 (3.7)	27.3 (3.9)	27.0 (3.6)
Weight (kg)	69.7 (12.3)	70.0 (12.9)	69.2 (11.8)	84.5 (12.9)	86.0 (13.5)	83.6 (12.5)
Height (cm)	163.1 (6.2)	164.4 (6.2)	162.4 (6.1)	176.5 (6.7)	177.6 (6.7)	175.8 (6.6)
WC (cm)	82.4 (11.1)	81.0 (11.1)	82.8 (10.9)	94.8 (10.1)	94.1 (10.3)	95.1 (10.0)
HC (cm)	102.1 (9.1)	101.7 (9.3)	102.0 (8.9)	102.6 (6.7)	103.0 (6.8)	102.4 (6.6)
ABSI	73.3 (4.8)	72.4 (4.6)	73.8 (4.9)	79.2 (4.0)	78.2 (3.9)	79.9 (4.0)
HI	64.3 (2.4)	64.1 (2.4)	64.4 (2.4)	49.1 (1.6)	49.0 (1.6)	49.2 (1.6)
BMI category^b,^***						
18.5 to < 25 kg/m^2^	57,521 (45.9)	19,175 (50.6)	33,634 (44.6)	34,326 (29.9)	13,116 (29.4)	21,210 (30.2)
25 to < 30 kg/m^2^	45,845 (36.5)	12,353 (32.6)	28,941 (38.3)	58,546 (51.0)	22,322 (50.1)	36,224 (51.6)
30 to < 45 kg/m^2^	22,069 (17.6)	6389 (16.8)	12,916 (17.1)	21,888 (19.1)	9155 (20.5)	12,733 (18.1)
Body shape phenotypes^b,^***						
Pear	33,369 (26.6)	11,402 (30.1)	18,775 (24.9)	30,990 (27.0)	14,170 (31.8)	16,820 (24.0)
Slim	28,836 (23.0)	10,566 (27.9)	15,501 (20.5)	35,393 (30.8)	16,339 (36.6)	19,054 (27.2)
Wide	36,548 (29.1)	8711 (23.0)	24,404 (32.3)	30,391 (26.5)	8613 (19.3)	21,778 (31.0)
Apple	26,682 (21.3)	7238 (19.1)	16,811 (22.3)	17,986 (15.7)	5471 (12.3)	12,515 (17.8)
Platelet parameters^c,^***						
Platelet count (10^9^/L)	259 (166 to 403)	262 (166 to 413)	257 (166 to 398)	234 (150 to 365)	238 (154 to 367)	231 (148 to 363)
MPV (fL)	9.31 (7.47 to 11.59)	9.41 (7.55 to 11.72)	9.26 (7.44 to 11.53)	9.20 (7.41 to 11.42)	9.24 (7.45 to 11.44)	9.18 (7.38 to 11.41)
PDW (%)	16.41 (15.47 to 17.41)	16.39 (15.46 to 17.38)	16.42 (15.48 to 17.41)	16.54 (15.57 to 17.58)	16.52 (15.55 to 17.55)	16.56 (15.58 to 17.59)

*ABSI* a body shape index (cutoffs ≥ 73 for women, ≥ 80 for men); *Apple* large-ABSI–small-HI; *BMI* body mass index; *HC *hip circumference; *HI* hip index (cutoffs ≥ 64 for women, ≥ 49 for men); *MPV* mean platelet volume; *PDW* platelet distribution width (coefficient of variation of platelet size); *Pear* small-ABSI–large-HI; *Post-MP* post-menopausal women; *Pre-MP* pre-menopausal women; *Slim* small-ABSI–small-HI; *WC *waist circumference; *Wide* large-ABSI–large-HI

^a^Mean (standard deviation)

^b^Count (percentage from total per column)

^c^Geometric mean (reference range)

***Pairwise comparisons for men vs. women overall, Post-MP vs. Pre-MP women, and men ≥ 52 years vs. < 52 years were performed with *t* test for continuous variables (log-transformed platelet parameters) or χ^2^-test for categorical variables. All comparisons were significant at p < 1 × 10^–6^

### Associations of body size with platelets

**Fig. 1:**
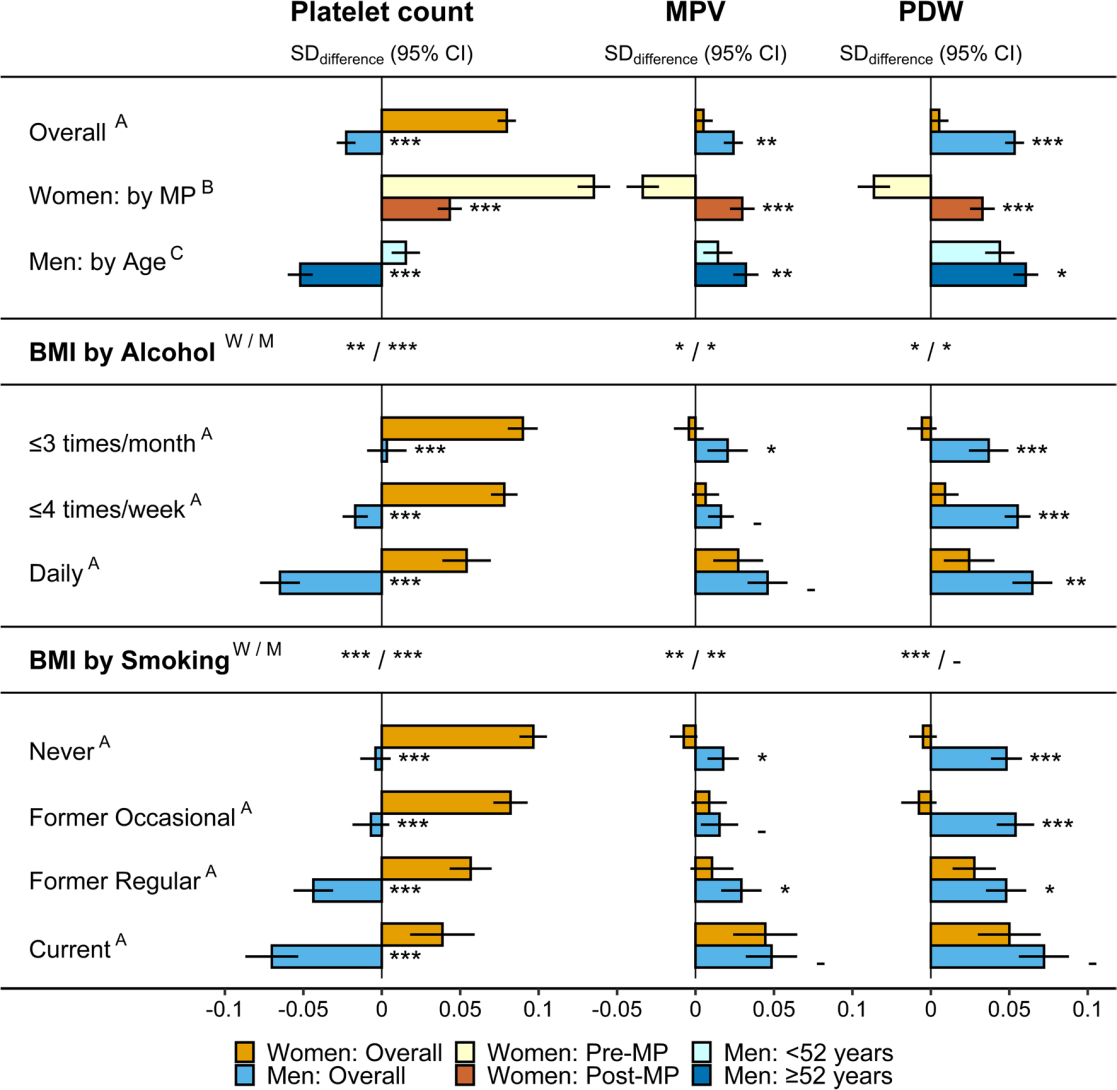
Associations of BMI with platelet parameters. *BMI *body mass index; *CI *confidence interval; *MPV *mean platelet volume; *PDW *platelet distribution width; *Post-MP *post-menopausal women; *Pre-MP *pre-menopausal women; *SD *standard deviation. SD_differences_ (95% CI) in platelet parameters per one SD increment of BMI from multivariable linear regression models including each platelet parameter as an outcome variable (sex-specific *z*-scores, following log-transformation) and BMI, ABSI, and HI as exposure variables (sex-specific *z*-scores), with adjustment for height, age, weight change (last year), smoking status (except for subgroups by smoking status), alcohol consumption (except for subgroups by alcohol consumption), physical activity, Townsend deprivation index, region of the assessment centre, time of blood collection, fasting time, use of nonsteroidal anti-inflammatory drugs, paracetamol use, menopausal status (women overall), hormonal replacement therapy use (women overall and Post-MP), oral contraceptives use and age at the last live birth (all women). Numerical values are shown in Additional file 1: Table S3. ^A ^*p* value for the interaction term of BMI with sex, from a model including women (reference) and men (separately for subgroups by alcohol consumption and smoking status), with adjustment for ABSI, HI, covariates (except female-specific), and including an interaction term of age with sex, to account for potential differences by menopausal status in women. ^B ^*p* value for the interaction term of BMI with menopausal status, from a model including Pre-MP (reference) and Post-MP women, with adjustment for ABSI, HI, and covariates. ^C ^*p* value for the interaction term of BMI with age (continuous), from a model including all men, with adjustment for ABSI, HI, and covariates (except female-specific). ^W/M^ *p* value (women/men) derived from likelihood ratio tests comparing models with and without an interaction term of BMI with either alcohol consumption or smoking status, separately for women and men, with adjustment for ABSI, HI, and covariates. *p* values  **-**  *p* ≥ 0.05; *p < 0.05; **p < 0.001; ***p < 1 × 10^–6^

BMI was associated positively with platelet count only in women, with a stronger association in pre-menopausal compared to post-menopausal women, but was associated inversely in men overall, with a weak positive association in younger men and a strong inverse association in older men (Fig. 1). The association of BMI with platelet count was positive for all subgroups according to alcohol consumption and smoking status in women, but was strongest for low alcohol consumption (≤ 3 times/month) and for never smokers and was weakest for daily alcohol consumption and current smoking (Fig. 1). In men, there was no evidence for association of BMI with platelet count for low alcohol consumption and in never smokers, but a strong inverse association for daily alcohol consumption and in current smokers (Fig. 1).

For platelet size, BMI was associated positively with MPV and more strongly with PDW in men, especially in older men. No evidence for association in women overall, however, was accounted for by an inverse association in pre-menopausal and a positive association in post-menopausal women, similarly for MPV and PDW (Fig. 1). In subgroups according to alcohol consumption and smoking status, BMI showed the strongest positive associations with MPV in women and men and with PDW in women for daily alcohol consumption and in current smokers, but there was little evidence for heterogeneity of the association of BMI with PDW in men (Fig. 1).

### Association of body shape with platelets

**Fig. 2:**
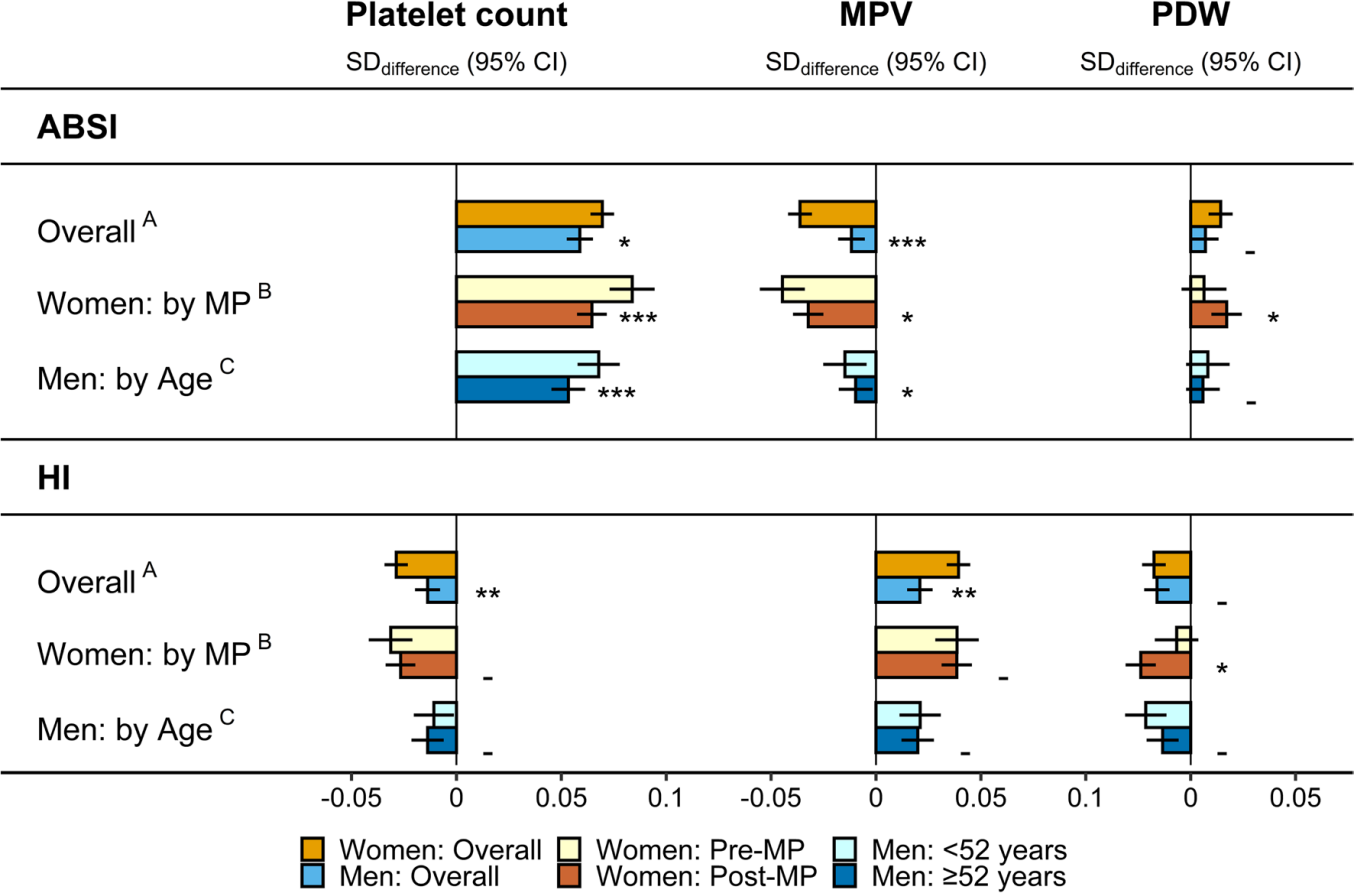
Associations of ABSI and HI with platelet parameters. *ABSI *a body shape index; *CI *confidence interval; *HI *hip index; *MPV* mean platelet volume; *PDW* platelet distribution width; *Post-MP* post-menopausal women; *Pre-MP* pre-menopausal women; *SD *standard deviation. SD_differences_ (95% CI) in platelet parameters per one SD increment of ABSI or HI from multivariable linear regression models including each platelet parameter as an outcome variable (sex-specific *z*-scores, following log-transformation) and body mass index (BMI), ABSI, and HI as exposure variables (sex-specific z-scores), with adjustment for height, age, weight change (last year), smoking status, alcohol consumption, physical activity, Townsend deprivation index, region of the assessment centre, time of blood collection, fasting time, use of nonsteroidal anti-inflammatory drugs, paracetamol use, menopausal status (women overall), hormonal replacement therapy use (women overall and Post-MP), oral contraceptives use and age at the last live birth (all women). Numerical values are shown in Additional file 1: Table S4. ^A ^*p* values for the interaction terms of ABSI and HI with sex, from a model including women (reference) and men, with adjustment for BMI, covariates (except female-specific), and including an interaction term of age with sex, to account for potential differences by menopausal status in women. ^B ^*p* values for the interaction terms of ABSI and HI with menopausal status, from a model including Pre-MP (reference) and Post-MP women, with adjustment for BMI and covariates. ^C ^*p* values for the interaction terms of ABSI and HI with age (continuous), from a model including all men, with adjustment for BMI and covariates (except female-specific). *p* values **-** *p* ≥ 0.05; *p < 0.05; **p < 0.001; *** *p* < 1 × 10^–6^

ABSI was associated positively with platelet count, with little difference between women and men but somewhat stronger in pre-menopausal women and younger men compared to post-menopausal women and older men, correspondingly (Fig. 2). HI was associated more weakly inversely with platelet count, mainly in women. Opposite to platelet count, ABSI was associated inversely and HI positively with MPV, mainly in women, while associations with PDW were directionally consistent with associations with platelet count (positive for ABSI and inverse for HI), but were very weak (Fig. 2). Neither alcohol consumption nor smoking status influenced materially the associations of ABSI and HI with platelet parameters (Additional file 1: Fig. S1).

**Fig. 3:**
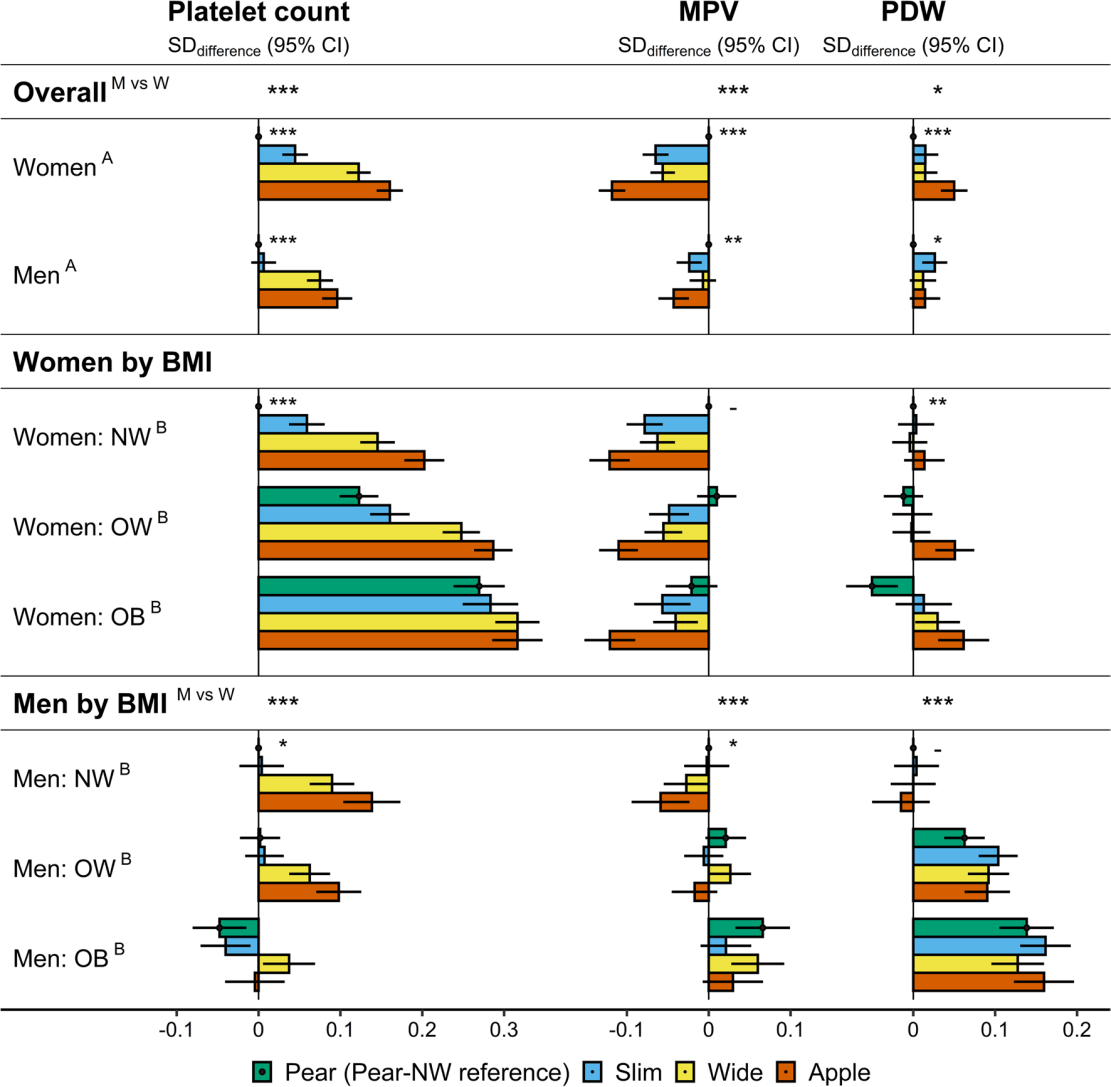
Associations of body shape phenotypes with platelet parameters. *ABSI* a body shape index (cutoffs ≥ 73 for women; ≥ 80 for men); *Apple *large-ABSI–small-HI; *BMI* body mass index; *CI* confidence interval; *HI* hip index (cutoffs ≥ 64 for women; ≥ 49 for men); *MPV* mean platelet volume; *NW* normal weight (BMI ≥ 18.5 to BMI < 25 kg/m^2^); *OB* obese (BMI ≥ 30 to BMI < 45 kg/m^2^); *OW* overweight (BMI ≥ 25 to BMI < 30 kg/m^2^); *PDW* platelet distribution width; *Pear* small-ABSI–large-HI; *SD* standard deviation; *Slim* small-ABSI–small-HI; *Wide* large-ABSI–large-HI. SD_differences_ (95% CI) in platelet parameters compared to the reference category from multivariable linear regression models including each platelet parameter as an outcome variable (sex-specific z-scores, following log-transformation) and as exposures, ABSI-by-HI (“pear” reference) and BMI categories (A), or BMI-by-ABSI-by-HI (“pear” NW reference) (B), with adjustment for height, age, weight change (last year), smoking status, alcohol consumption, physical activity, Townsend deprivation index, region of the assessment centre, time of blood collection, fasting time, use of nonsteroidal anti-inflammatory drugs, paracetamol use, and for women, menopausal status, hormonal replacement therapy use, oral contraceptives use, and age at the last live birth. Numerical values are shown in Additional file 1: Table S5. ^A/B^ *p* values for body shape overall (next to “pear”) or for heterogeneity of the associations with body shape according to BMI category (next to “pear-NW”) from likelihood ratio tests, separately for women and men, comparing fully adjusted models including BMI categories with and without ABSI-by-HI (A), or the fully adjusted additive model, including ABSI-by-HI and BMI categories, with the interaction model, including BMI-by-ABSI-by-HI (B). ^M vs W^ *p* values for men vs. women from likelihood ratio tests comparing models including women and men with and without an interaction term of ABSI-by-HI with sex (row Overall) or of BMI-by-ABSI-by-HI with sex (row Men), adjusted for covariates (except female-specific), and including an interaction term of age with sex. *p* values - *p* ≥ 0.05; *p < 0.05; **p < 0.001; ***p < 1*10^–6^

Corresponding to the predominant positive association with ABSI, the highest platelet counts were for “apple” and “wide” phenotypes in women and men (using “pear” phenotype as reference), but “slim” phenotype showed slightly higher platelet counts only in women (Fig. 3). On the contrary, MPV was lowest for “apple” and highest for “pear” phenotype, with both “wide” and “slim” phenotypes showing similar intermediate levels, mainly in women. In addition, in women, PDW was higher only for “apple” phenotype (Fig. 3).

Within BMI categories, the associations of body shape phenotypes with platelet count were stronger for normal weight and overweight BMI in both women and men, but were considerably weaker for obese BMI (Fig. 3, see also Additional file 1: Table S5 for comparisons between “apple” and “pear” phenotype within each BMI category). The association pattern with MPV, however, was maintained for all BMI categories in women, while the difference in PDW between “apple” and “pear” phenotype was largest in obese women (Fig. 3).

### Correlation of platelet count and size with metabolic and inflammatory biomarkers

**Fig. 4:**
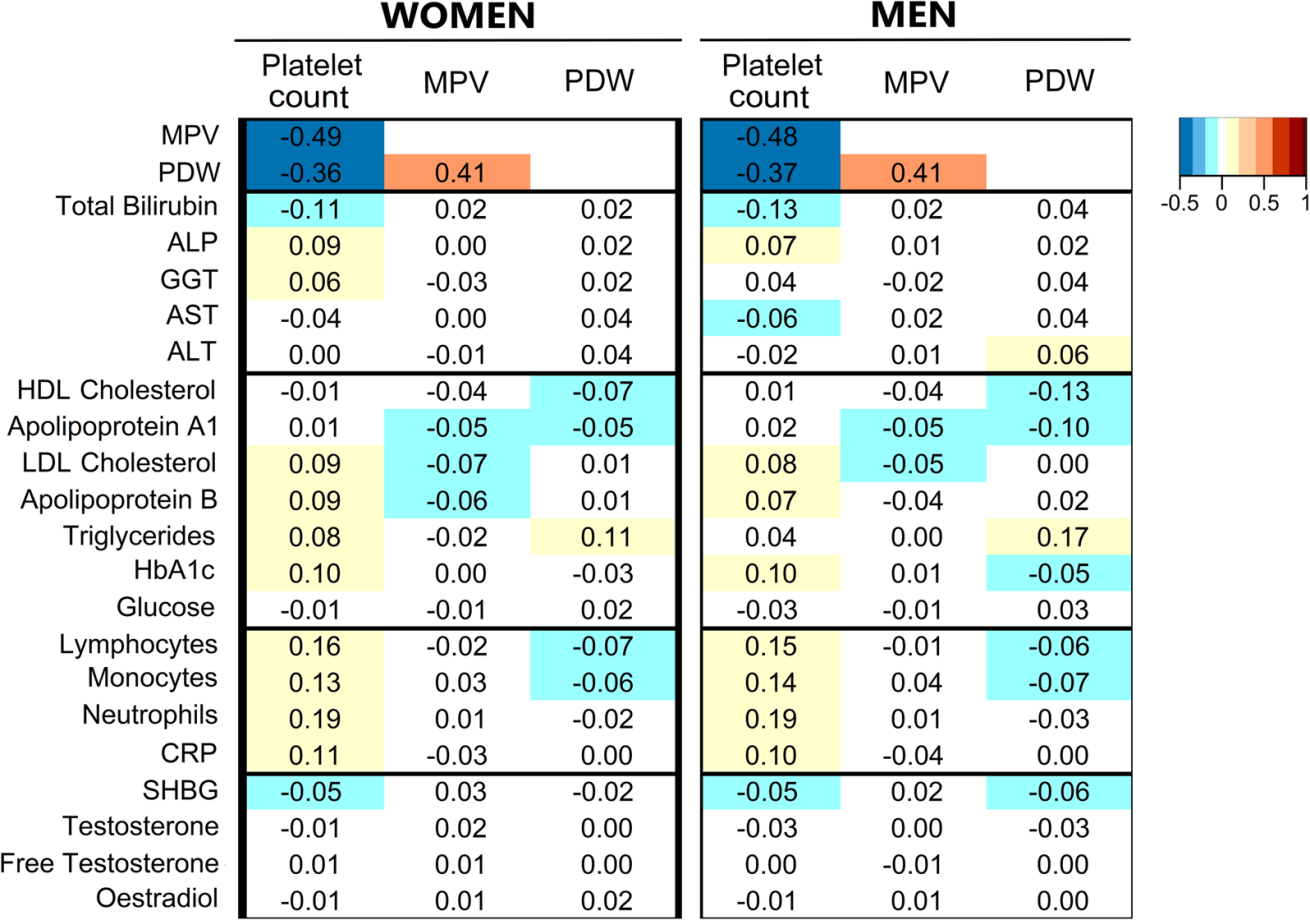
Correlation of platelet parameters with liver function tests, metabolic, inflammatory, and sex steroid biomarkers. *ALP* alkaline phosphatase; *ALT* alanine aminotransferase; *AST* aspartate aminotransferase; *CRP* C-reactive protein; *GGT* gamma-glutamyltransferase; *HbA1c* haemoglobin A1c (glycated haemoglobin); *HDL* high-density lipoprotein; *LDL* low-density lipoprotein; *MPV* mean platelet volume; *PDW* platelet distribution width; *SHBG* sex hormone binding globulin. Values represent partial Pearson correlation coefficients with adjustment for body mass index, a body shape index, hip index, height, age, weight change (last year), smoking status, alcohol consumption, physical activity, Townsend deprivation index (continuous), time of blood collection (continuous), fasting time (continuous), use of nonsteroidal anti-inflammatory drugs, paracetamol use, and for women, menopausal status, hormone replacement therapy use, oral contraceptives use, and age at the last live birth. Correlation coefficients were calculated in a subset of participants with available all biomarker measurements (94,285 (75.2%) women; 87,702 (76.4%) men)

Independent of body size, body shape, and covariates, platelet count was correlated substantially inversely with both MPV and PDW, which were correlated positively with each other (Fig. 4). Platelet count was correlated weakly positively with all leucocyte subtypes but most strongly with neutrophils and, to some extent, with C-reactive protein and glycated haemoglobin, but weakly inversely with total bilirubin. MPV showed no material associations with biomarkers or leucocyte counts and PDW was only correlated weakly positively with triglycerides and weakly inversely with high-density lipoprotein cholesterol and apolipoprotein A1, mainly in men. Neither platelet count nor platelet size showed any material correlations with testosterone, oestradiol, or SHBG.

### Sensitivity analyses

Examining BMI categories and tertiles of ABSI and HI, there were no indications for U-shaped associations or for major deviations from linearity. Suggestive of a plateau (similar associations for overweight and obese compared to normal weight) were only the positive associations of BMI with platelet count in post-menopausal women and in younger men, while MPV was lower only for obese and not for overweight pre-menopausal women and was higher only for obese young men (Additional file 1: Fig. S2). Restricting the data set to participants with stable weight (Additional file 1: Fig. S3) or without medication use (Additional file 1: Fig. S4) made no material difference to association estimates. Omitting all covariates, however, resulted in slightly stronger positive associations of BMI with platelet count in women and in younger men but a weaker inverse association in older men (Additional file 1: Fig. S5). The unadjusted association estimates of ABSI with platelet count were also somewhat weaker in women and in older men.

## Discussion

In the middle-aged UK Biobank cohort, excluding participants with prevalent cancer, endocrine and metabolic conditions, and severe illnesses, we report directional sex-specific differences in the associations of obesity indices with platelet count and platelet size.

Sex differences in platelet count, higher in women compared to men, are well established [30–32] and suggest an involvement of sex steroids in platelet production. This is supported further by the sex differences in the associations of BMI with platelet count that we have shown, positive in women but inverse in men. Although platelet count was lower in post-menopausal compared to pre-menopausal women and in older compared to younger men, in accordance with smaller scale studies [30, 31], sex differences in platelet count and its associations with BMI were retained for all ages. However, post-menopausal women not using HRT, which have lower circulating oestradiol than men [26], maintained higher platelet count than men and a positive association with BMI and, notably, platelet count was not correlated with circulating sex steroids. Our results, therefore, suggest, that the response of platelet production to BMI-related factors is set differently in women and men, most likely during intrauterine development or during puberty, but does not depend further on the levels of circulating sex steroids in middle adulthood. Nevertheless, the association between BMI and platelet count was shifted towards the inverse direction for both post-menopausal women and older men, suggesting that an age-related factor alters the influence of BMI on platelet count similarly in women and men.

Our study, therefore, is the first to show that competing BMI-related factors influence platelet count, one group contributing to a positive association and subject to sex differences, and another contributing to an inverse association and related to age. Our findings would, therefore, explain the discrepancies in published studies for the association of obesity with platelet count. The direction of the association would be determined by the balance between the opposed BMI-related factors, which would depend on the proportion of women and men in the study (for joint association estimates) and on the age of study participants. Thus, studies examining separately women and men with mean age around 40 years, have reported a positive association of BMI with platelet count in women but no evidence for association in men [15, 16]. However, a study including younger participants, 80% of whom were men but with an average age around 30 years, has reported higher platelet count for obese compared to normal weight individuals [14]. This is compatible with the weak positive association of BMI with platelet count that we have shown for younger men.

The positive association of BMI with platelet count matches the positive associations of BMI with leucocyte subtype counts [12]. This is compatible with their coordinated function in inflammation, especially for neutrophils, as platelet–neutrophil interactions are required for neutrophil activation and recruitment to inflamed tissues [33, 34]. Therefore, inflammatory cytokines could be responsible for the positive association of BMI with platelet count. Correspondingly, animal studies have shown that obesity, induced by high fat diet, results in increased production of inflammatory cytokines, bone marrow hyperplasia, and leucocytosis [35–37]. It is less clear, however, whether these changes are coordinated with thrombocytosis, as some factors such as cytokine receptor-like factor 3 (CRLF3) regulate platelet formation in the hematopoietic bone marrow compartment without affecting leucocyte lineages [38]. Another factor contributing to platelet production is thrombopoietin, which is higher in obesity, at least in women [39]. Cortisol could also be contributing to a positive association with BMI, as glucocorticoids promote the responsiveness to thrombopoietin receptor agonists [40] and contribute to granulocytosis by delaying apoptosis and increasing the release of polymorphonuclear cells from the bone marrow and from the endothelial surface [41]. Patients with Cushing’s syndrome have higher platelet count compared to healthy controls and an overall state of hypercoagulability [42], as well as higher neutrophil count, which decrease following treatment [43].

The inverse association of BMI with platelet count is most likely related to liver damage and fibrosis resulting from liver fat infiltration, which in more extreme cases leads to non-alcoholic fatty liver disease (NAFLD) [44] and would further be aggravated by liver damage related to older age, high alcohol consumption, and smoking. Low platelet count is, indeed, a hallmark of fibrosis associated with cirrhosis and gradually decreases for several years prior to the overt clinical presentation of the disease [45]. Correspondingly, platelet count is included in most non-invasive liver fibrosis indices [46]. Smoking can also contribute to the development of liver fibrosis [47], in addition to high alcohol consumption [48], thus explaining why both shifted the association of BMI with platelet count towards the inverse direction. Female sex and oestrogens, however, show an apparent protective effect against NAFLD-related fibrosis [49], which could explain why women are more likely than men to balance the opposing BMI-related factors in the positive direction and why the positive association with BMI was strongest in pre-menopausal women.

Platelet size and platelet count were correlated inversely, in accordance with small scale studies [17], and showed associations with BMI in opposite directions. This is in accordance with opposite changes, a reduction of platelet count and an increase in MPV, after bariatric surgery [50, 51] and in association with liver fibrosis [45, 52]. The association of BMI with MPV and PDW, however, was inverse only for pre-menopausal women, but was positive for post-menopausal women, as well as for men. This could not have been identified in small scale studies combining both sexes and all ages, which have reported higher MPV and PDW for obesity [13, 17, 53]. Given that large and variable platelet size is indicative of platelet activation and thrombosis [9, 10], our findings of higher MPV and PDW in obese post-menopausal women and men are compatible with higher risk of thrombosis and cardiovascular diseases in these groups [54].

Another possibility for a trade-off between platelet size and count is suggested by the mechanism of platelet formation. Megakaryocytes in bone marrow, derived from haematopoietic stem cells, form platelets by extending long cytoskeletal processes and releasing circular-preplatelets in the circulation. Preplatelets evolve into barbell-shaped proplatelet intermediates, which undergo fission and form two smaller size mature platelets [55]. Megakaryocyte maturation and formation of proplatelets depend heavily on actin and microtubule cytoskeletons and mutations in some of the cytoskeletal proteins are associated with both large platelet size and reduced platelet number [56]. RNA processing is another mechanism controlling platelet formation. Deletion of the RNA-binding protein serine–arginine-rich splicing factor 3 (SRSF3) in mice results in arrest of megakaryocyte maturation, with sequestration of mRNAs in the megakaryocyte nucleus and production of a smaller number of dysfunction platelets with abnormally large size [57]. CRLF3 also plays a role in the maturation of proplatelets, with CRLF3 deficiency contributing to thrombocytopenia due to inefficient thrombopoiesis, with preplatelets, which are larger than the mature platelets, circulating for longer and being destroyed in the spleen before managing to mature into platelets [38].

While the associations of BMI with platelet count and size showed clear sex and age differences and a similar association pattern for MPV and PDW, the association of body shape with platelet count showed small sex differences and minimal age differences. Body shape was also associated only with MPV, in opposite directions to the associations with platelet count, but mainly in women and there was little evidence for associations of body shape with PDW. The predominant positive association of ABSI with platelet count in women and men, with similarly higher levels in both “apple” and “wide” compared to “pear” phenotype, resembled the association patterns of body shape with monocyte and neutrophil counts and C-reactive protein [12], but unlike them, the associations of ABSI with platelet count was weaker for obese BMI. Cortisol is among the main factors determining body shape [58] that could contribute to a positive association with ABSI, as well as with BMI, similarly for platelet and neutrophil counts. It is unclear, however, what factors could explain the associations of body shape with platelet size.

A major strength of our study is the very large sample size compared to previous reports, which enabled us to examine in more detail subgroups by menopausal status in women and by age in men. We have also applied rigorous exclusion criteria, which has minimised the possibility for reverse causality. We were able to adjust for major lifestyle and reproductive factors, which clearly influenced the association estimates, and we have thus minimised confounding. Our study was not affected by bias from self-reporting, as anthropometric measurements were obtained by trained personnel according to standardised procedures. Measurement errors were also minimised by the standardised approach to blood sample collection and storage, the use of a single type of automatic blood count analyser for all samples, and the use of systematic quality control for all biomarker measurements.

Our study, admittedly, has several limitations. Most importantly, we did not have information on haemocoagulation and platelet activity and could not evaluate to what extent differences in platelet count and size contributed to different thrombotic states. We were unable to examine early or late adulthood, because UK Biobank is a middle-aged cohort. We could not examine underweight, or severe obesity, or ethnic variations either, due to limited numbers in these categories. A misclassification of medication use or self-reported disease status at recruitment is also possible, which may have affected the exclusions. Our study was also cross-sectional and we could not assess temporality. Last, UK Biobank participants have a healthier lifestyle and are not representative of the overall UK population [59].

## Conclusions

Competing BMI-related factors determine the associations of BMI with platelet count. Factors with sexually dimorphic actions contribute to a positive association, more prominent in women compared to men, and age-dependent factors, aggravated by smoking and alcohol consumption, contribute to an inverse association. Inflammatory cytokines, thrombopoietin, and cortisol may contribute to the positive association with BMI, while the inverse association would likely be explained by liver damage and fibrosis. Associations of body shape with platelet count, predominantly positive with waist size and with minor sex differences, resemble the association patterns of body shape with inflammatory biomarkers (Creactive protein, neutrophil and monocyte counts). Platelet size is correlated inversely with platelet count, both for MPV and PDW, and shows associations with BMI in opposite direction to platelet count but only MPV is associated with body shape and only in women.

## Perspectives and significance

Our study shows that, in addition to the constitutively higher platelet count and size in women compared to men, there are directional differences in the associations of obesity indices with platelet parameters. In women, especially in pre-menopausal women, appear more prominent obesity-related factors stimulating platelet synthesis, with more complete maturation of platelet particles and smaller platelet size. In men, especially in older men, as well as in smokers and in daily alcohol consumers, apparently gain more prominence restrained platelet maturation and platelet destruction, facilitated by obesity-related liver damage. It is important, therefore, when evaluating platelet status in clinical practice to consider how sex, age, menopause, lifestyle factors, and obesity are balanced for each individual and, in research, to consider how these factors are balanced for a given data set. It would further be important to evaluate in future studies the impact of obesity on the diagnostic value of platelet-based indices, such as the platelet-to-leucocyte ratio or the non-invasive indices of liver fibrosis. Sex differences in platelet–neutrophil co-operations and in platelet contributions to obesity-related complications, such as atherosclerosis and cancer development, should also be examined.

## Supplementary Information

**Additional file 1: Table S1** Flow chart of UK Biobank participants in the study. **Table S2** Lifestyle and reproductive characteristics of study participants. **Table S3** Associations of BMI with platelet parameters. **Table S4 **Associations of ABSI and HI with platelet parameters. **Table S5** Associations of body shape phenotypes with platelet parameters. **Figure S1** Associations of ABSI and HI with platelet parameters (subgroups by alcohol consumption and smoking status). **Figure S2** Associations of anthropometric index categories with platelets. **Figure S3** Associations of anthropometric indices with platelets in participants with stable weight. **Figure S4** Associations of anthropometric indices with platelets in participants without NSAID or paracetamol use. **Figure S5** Unadjusted associations of anthropometric indices with platelets.

## Data Availability

The data supporting the findings of the study are available to bona fide researchers upon approval of an application to the UK Biobank (https://www.ukbiobank.ac.uk/researchers/) and a material transfer agreement.

## Abbreviations

ABSI: A body shape index
BMI: Body mass index
CI: Confidence interval
CRLF3: Cytokine receptor-like factor 3
HC: Hip circumference
HI: Hip index
HR: Hazard ratio
MPV: Mean platelet volume
NAFLD: Non-alcoholic fatty liver disease
NHANES: National Health and Nutrition Examination Survey
NSAID: Nonsteroidal anti-inflammatory drugs
PDW: Platelet distribution width
SD: Standard deviation
WC: Waist circumference
