# Supplementary material for: Sex differences in the associations of body size and body shape with platelets in the UK Biobank cohort
Source: Biol Sex Differ. 2023 Feb 22;14:12. doi: 10.1186/s13293-023-00494-y (PMC9945692; doi:10.1186/s13293-023-00494-y)
Supplement: Supplementary file 1 — Additional file 1: Table S1 Flow chart of UK Biobank participants in the study. Table S2 Lifestyle and reproductive characteristics of study participants. Table S3 Associations of BMI with platelet parameters. Table S4 Associations of ABSI and HI with platelet parameters. Table S5 Associations of body shape phenotypes with platelet parameters. Figure S1 Associations of ABSI and HI with platelet parameters (subgroups by alcohol consumption and smoking status). Figure S2 Associations of anthropometric index categories with platelets. Figure S3 Associations of anthropometric indices with platelets in participants with stable weight. Figure S4 Associations of anthropometric indices with platelets in participants without NSAID or paracetamol use. Figure S5 Unadjusted associations of anthropometric indices with platelets. [file 13293_2023_494_MOESM1_ESM.pdf]

Sex differences in the associations of body size and body shape with platelets in the UK Biobank cohort

Sofia Christakoudi, Konstantinos K. Tsilidis, Evangelos Evangelou, Elio Riboli

Additional Tables

Table S1 Flow chart of UK Biobank participants in the study 2
Table S2 Lifestyle and reproductive characteristics of study participants 5
Table S3 Associations of BMI with platelet parameters 7
Table S4 Associations of ABSI and HI with platelet parameters 9
Table S5 Associations of body shape phenotypes with platelet parameters 11

Additional Figures

Fig. S1 Associations of ABSI and HI with platelet parameters (subgroups by alcohol consumption and smoking status) 13
Fig. S2 Associations of anthropometric index categories with platelets 15
Fig. S3 Associations of anthropometric indices with platelets in participants with stable weight 17
Fig. S4 Associations of anthropometric indices with platelets in participants without NSAID or paracetamol use 19
Fig. S5 Unadjusted associations of anthropometric indices with platelets 21

References 23

Table S1 Flow chart of UK Biobank participants in the study

	Exclusions	Women	Men
	**Total:**	**273,328**	**229,085**
1.	Ethnic background (restricted to self-reported white) [a]	15,936	13,862
2.	Anthropometry missing or extreme (ABSI or HI missing; height <130 cm; WC <50 or >160 cm; BMI <18.5 or ≥45 kg/m^2) [a]	4758	2372
3.	Age restrictions (age <40 or >70 years)	2	9
4.	Genetic & self-reported sex mismatch or sex chromosome aneuploidy [a]	386	396
5.	Pregnant or unknown at enrolment [a]	293	0
6.	Prevalent cancer at enrolment [b]	21,844	12,182
7.	Cancer or death within two years post enrolment [b]	3464	4078
8.	Diabetes at enrolment [a]	6828	12,349
9.	Thyroid-related illness [a]	19,233	3245
10.	Pituitary, adrenal, other endocrine illness [a]	432	225
11.	Liver-related illness or kidney failure [a]	1713	1923
12.	Inflammatory bowel disease [a]	1707	1442
13.	Chronic respiratory illness [a]	4678	5329
14.	Haematological disorders [c]	3070	1149
15.	Thromboembolic disorders [d]	4818	3520
16.	Stroke [e]	1728	2835
17.	Heat attack or failure [f]	2898	8618
18.	Lipid-lowering drugs [a]	13,853	20,543
19.	Anti-hypertensive drugs [a]	16,528	14,382
20.	Current HRT or OC use at enrolment [a]	16,259	0
21.	Glucocorticoids (oral) [a]	824	646
22.	Anticoagulants [g]	125	345
23.	Blood sample >28 days post enrolment [a]	1	0
24.	Missing all platelet measurements	6515	4875
	**Total excluded:**	**147,893**	**114,325**
	**%** (from total available)	**54.1**	**49.9**

**WOMEN** **125,435**	
Alcohol: ≤3 times/month	39,662 (31.6)
Alcohol: ≤4 times/week	63,804 (50.9)
Alcohol: Daily	21,969 (17.5)
Smoking: Never	55,926 (44.6)
Smoking: Former occasional	36,310 (28.9)
Smoking: Former regular	22,561 (18.0)
Smoking: Current	10,638 (8.5)
Stable Weight:	68,521 (54.6)
No Medication:	78,708 (62.7)

**MEN** **114,760**	
Alcohol: ≤3 times/month	21,648 (18.9)
Alcohol: ≤4 times/week	63,748 (55.5)
Alcohol Daily:	29,364 (25.6)
Smoking Never:	42,770 (37.3)
Smoking: Former occasional	31,879 (27.8)
Smoking: Former regular	25,904 (22.6)
Smoking Current:	14,207 (12.4)
Stable Weight:	75,565 (65.8)
No Medication:	82,691 (72.1)

**Pre-MP** **37,917** (30.2)	**Post-MP** **75,491** (60.2)	**40 to <52 years** **44,593** (38.9)	**52 to <70 years** **70,167** (61.1)
Stable Wt: 19,888 (52.5)	Stable Wt: 42,836 (56.7)	Stable Wt: 27,240 (61.1)	Stable Wt: 48,325 (68.9)
No Medic: 21,987 (58.0)	No Medic: 49,685 (65.8)	No Medic: 31,202 (70.0)	No Medic: 51,489 (73.4)

ABSI – a body shape index; **BMI** – body mass index; **HI** – hip index; **HRT** – hormone replacement therapy; **No Medication** – no nonsteroidal anti-inflammatory drugs or paracetamol; **OC** – oral contraceptives; **Pre-MP** – pre-menopausal; **Post-MP** – post-menopausal; **WC** – waist circumference.

Values represent number of participants (% from total included per sex or group) and include the imputations of missing values with the median sex-specific category. The exclusion criteria were applied sequentially in the displayed order, such that each excluded individual was counted only once. Specific fields used to define the exclusions are listed below:

[a] – for filed names and lists of disorders see the legend of Supplementary Figure S1 in [ref. 12], for list of medications see Supplementary Table S1 in [ref. 12].

[b] – for definition of prevalent and incident cancer cases and deaths see [ref. 23].

[c] – exclusions comprised haematological disorders self-reported at enrolment from Fields [20002-0.0…33] “*Non-cancer illness code, self-reported*”, including the following codes (as listed in UK Biobank Coding 6):

1327 low platelets/platelet disorder
1328 haemophilia
1330 iron deficiency anaemia
1331 pernicious anaemia
1332 aplastic anaemia
1339 sickle cell disease
1340 thalassaemia
1438 polycythaemia vera
1445 clotting disorder/excessive bleeding
1446 anaemia
1447 pancytopenia
1448 neutropenia/lymphopenia
1449 myeloproliferative disorder
1450 monoclonal gammopathy/not myeloma
1451 hereditary/genetic haematological disorder
1546 essential thrombocytosis
1658 myelofibrosis

Note from UK Biobank: *If the participant was uncertain of the type of illness they had had, then they described it to the interviewer (a trained nurse) who attempted to place it within the coding tree. If the illness could not be located in the coding tree, then the interviewer entered a free-text description of it. These free-text descriptions were subsequently examined by a doctor and, where possible, matched to entries in the coding tree. Free-text descriptions which could not be matched with very high probability have been marked as "unclassifiable"*.

[d] – exclusions comprised thromboembolic disorders self-reported at enrolment (as in [c]) with codes:

1067 peripheral vascular disease
1068 venous thromboembolic disease
1087 leg claudication/ intermittent claudication
1088 arterial embolism
1093 pulmonary embolism +/- dvt
1094 deep venous thrombosis (dvt)

[e] – exclusions comprised self-reported illness at enrolment (as in [c]) with codes:

1081 stroke
1082 transient ischaemic attack (tia)
1583 ischaemic stroke

[f] – exclusions comprised self-reported illness at enrolment (as in [c]) with codes:

1074 angina
1075 heart attack/myocardial infarction
1076 heart failure/pulmonary odema

[g] – information for self-reported anticoagulant medications at enrolment was obtained from Fields [20003-0.0…47] "*Treatment/medication code*" and included the following codes (as listed in UK Biobank Coding 4):

1140861506 calciparine 5000iu/0.2ml prefilled syringe
1140861568 minihep calcium 5000iu/0.2ml injection
1140861574 uniparin-ca 5000iu/0.2ml prefilled syringe
1140861578 monoparin-ca 5000iu/0.2ml injection
1140861584 fragmin 10,000iu/1ml injection
1140861588 enoxaparin
1140861594 clexane 20mg/0.2ml prefilled syringe
1140861602 innohep 5000iu/0.5ml injection amp
1140861604 logiparin 2500iu/0.21ml prefilled syringe
1140864956 subcutaneous heparin
1140877958 heparinoid+salicylic acid 0.2%/2% cream
1140877960 heparinoid+salicylic acid 0.2%/2% gel
1140881842 heparin
1140888204 dalteparin
1140888206 tinzaparin
1140888266 warfarin
1140910832 sodium warfarin
1140926360 alphaparin 3000iu/0.3ml prefilled syringe
1140926444 certoparin
1141171364 reviparin
1141171374 clivarine 1432iu/0.25ml prefilled syringe
1141189054 bemiparin

Table S2 Lifestyle and reproductive characteristics of study participants

	WOMEN			MEN		
	**Overall**	**Pre-MP**	**Post-MP**	**Overall**	**<52 years**	**≥52 years**
Cohort: n (% per sex)	125,435 (52.2)	37,917 (30.2)	75,491 (60.2)	114,760 (47.8)	44,593 (38.9)	70,167 (61.1)
**Weight change (last year)**: n (%)						
Lost weight	17,430 (13.9)	5602 (14.8)	10,014 (13.3)	14,790 (12.9)	6376 (14.3)	8414 (12.0)
Stable weight	66,473 (53.0)	19,275 (50.8)	41,627 (55.1)	73,480 (64.0)	26,371 (59.1)	47,109 (67.1)
Gained weight	39,484 (31.5)	12,427 (32.8)	22,641 (30.0)	24,405 (21.3)	10,977 (24.6)	13,428 (19.1)
Missing	2048 (1.6)	613 (1.6)	1209 (1.6)	2085 (1.8)	869 (1.9)	1216 (1.7)
**Smoking status**: n (%)						
Never	55,926 (44.6)	17,385 (45.9)	33,180 (44.0)	42,770 (37.3)	18,153 (40.7)	24,617 (35.1)
Former occasional	35,979 (28.7)	11,152 (29.4)	21,465 (28.4)	31,589 (27.5)	12,688 (28.5)	18,901 (26.9)
Former regular	22,561 (18.0)	5536 (14.6)	14,975 (19.8)	25,904 (22.6)	6996 (15.7)	18,908 (26.9)
Current	10,638 (8.5)	3775 (10.0)	5659 (7.5)	14,207 (12.4)	6681 (15.0)	7526 (10.7)
Missing	331 (0.3)	69 (0.2)	212 (0.3)	290 (0.3)	75 (0.2)	215 (0.3)
**Alcohol consumption**: n (%)						
≤3 times/month (low)	39,662 (31.6)	11,350 (29.9)	24,120 (32.0)	21,648 (18.9)	9438 (21.2)	12,210 (17.4)
≤4 times/week	63,742 (50.8)	20,905 (55.1)	36,869 (48.8)	63,689 (55.5)	26262 (58.9)	37,427 (53.3)
Daily	21,969 (17.5)	5647 (14.9)	14,462 (19.2)	29,364 (25.6)	8861 (19.9)	20,503 (29.2)
Missing	62 (<0.1)	15 (<0.0)	40 (0.1)	59 (0.1)	32 (0.1)	27 (<0.1)
**Low alcohol consumption in never smokers**: n (% from never smokers)						
Low Alc & Never Smk	20,771 (37.1)	6098 (35.1)	12,539 (37.8)	9816 (23.0)	4549 (25.1)	5267 (21.4)
**Daily alcohol consumption in current smokers**: n (% from current smokers)						
Daily Alc & Current Smk	2372 (22.3)	803 (21.3)	1312 (23.2)	4531 (31.9)	1903 (28.5)	2628 (34.9)
**Physical activity**: n (%)						
Inactive	19,415 (15.5)	6008 (15.8)	11,365 (15.1)	15,394 (13.4)	5689 (12.8)	9705 (13.8)
Moderately active	64,689 (51.6)	17,867 (47.1)	40,807 (54.1)	49,139 (42.8)	16,886 (37.9)	32,253 (46.0)
Active	41,018 (32.7)	13,978 (36.9)	23,127 (30.6)	49,996 (43.6)	21,929 (49.2)	28,067 (40.0)
Missing	313 (0.2)	64 (0.2)	192 (0.3)	231 (0.2)	89 (0.2)	142 (0.2)
**Townsend index** [#]						
Mean (SD)	-1.64 (2.85)	-1.41 (2.96)	-1.77 (2.79)	-1.56 (2.95)	-1.22 (3.09)	-1.78 (2.84)
Missing: n (%)	147 (0.1)	57 (0.2)	75 (0.1)	141 (0.1)	77 (0.2)	64 (0.1)
**Time of blood collection**: n (%)						
8 am to <12 am	32,186 (25.7)	11,112 (29.3)	17,943 (23.8)	31,893 (27.8)	13,399 (30.0)	18,494 (26.4)
12 am to <4 pm	51,795 (41.3)	14,025 (37.0)	32,984 (43.7)	41,557 (36.2)	14,425 (32.3)	27,132 (38.7)
4 pm to ≤8:15 pm	41,348 (33.0)	12,744 (33.6)	24,503 (32.5)	41,233 (35.9)	16,741 (37.5)	24,492 (34.9)
Missing	106 (0.1)	36 (0.1)	61 (0.1)	77 (0.1)	28 (0.1)	49 (0.1)
**Fasting time**: n (%)						
0-2 hours	34,206 (27.3)	12,001 (31.7)	18,976 (25.1)	32,002 (27.9)	14,155 (31.7)	17,847 (25.4)
3-4 hours	65,216 (52.0)	18,369 (48.4)	40,638 (53.8)	55,874 (48.7)	19,830 (44.5)	36,044 (51.4)
≥5 hours	26,008 (20.7)	7545 (19.9)	15,875 (21.0)	26,882 (23.4)	10,607 (23.8)	16,275 (23.2)
Missing	5	2	2	2	1	1
**NSAID**: n (%)						
No	96,525 (77.0)	27,381 (72.2)	60,208 (79.8)	92,199 (80.3)	35,045 (78.6)	57,154 (81.5)
Yes	28,004 (22.3)	10,291 (27.1)	14,736 (19.5)	21,458 (18.7)	9087 (20.4)	12,371 (17.6)
Missing	906 (0.7)	245 (0.6)	547 (0.7)	1103 (1.0)	461 (1.0)	642 (0.9)
**Paracetamol**: n (%)						
No	92,874 (74.0)	26,710 (70.4)	57,739 (76.5)	94,901 (82.7)	35,582 (79.8)	59,319 (84.5)
Yes	31,638 (25.2)	10,959 (28.9)	17,192 (22.8)	18,747 (16.3)	8552 (19.2)	10,195 (14.5)
Missing	923 (0.7)	248 (0.7)	560 (0.7)	1112 (1.0)	459 (1.0)	653 (0.9)
**Assessment region**: n (%)						
London	15,328 (12.2)	5040 (13.3)	9175 (12.2)	13,113 (11.4)	5180 (11.6)	7933 (11.3)
North-West	17,502 (14.0)	5127 (13.5)	10,543 (14.0)	17,272 (15.1)	6709 (15.0)	10,563 (15.1)
North-East	14,987 (11.9)	4354 (11.5)	9244 (12.2)	13,811 (12.0)	5371 (12.0)	8440 (12.0)
Yorkshire and Humber	19,367 (15.4)	5493 (14.5)	11,733 (15.5)	17,376 (15.1)	6528 (14.6)	10,848 (15.5)
West Midlands	10,051 (8.0)	2985 (7.9)	6150 (8.1)	10,399 (9.1)	4057 (9.1)	6342 (9.0)
East Midlands	8849 (7.1)	2485 (6.6)	5494 (7.3)	7951 (6.9)	2865 (6.4)	5086 (7.2)
South-East	12,058 (9.6)	3596 (9.5)	7217 (9.6)	10,872 (9.5)	4128 (9.3)	6744 (9.6)
South-West	12,159 (9.7)	3974 (10.5)	6967 (9.2)	10,651 (9.3)	4404 (9.9)	6247 (8.9)
Wales	5227 (4.2)	1676 (4.4)	3036 (4.0)	4823 (4.2)	1894 (4.2)	2929 (4.2)
Scotland	9907 (7.9)	3187 (8.4)	5932 (7.9)	8492 (7.4)	3457 (7.8)	5035 (7.2)

	WOMEN			MEN		
	Overall	Pre-MP	Post-MP	Overall	<52 years	≥52 years
**HRT use**: n (%)						
Never	89,467 (71.3)	37,198 (98.1)	43,756 (58.0)			
Former	35,673 (28.4)	646 (1.7)	31,584 (41.8)			
Missing	295 (0.2)	73 (0.2)	151 (0.2)			
**OC use**: n (%)						
Never	19,653 (15.7)	3716 (9.8)	14,319 (19.0)			
Former	105,551 (84.1)	34,133 (90.0)	61,056 (80.9)			
Missing	231 (0.2)	68 (0.2)	116 (0.2)			
**Age at the last live birth**: n (%)						
No live births	23,928 (19.1)	9462 (25.0)	12,582 (16.7)			
< 30 years	44,317 (35.3)	9097 (24.0)	29,960 (39.7)			
≥ 30 years	56,966 (45.4)	19,302 (50.9)	32,818 (43.5)			
Missing	224 (0.2)	56 (0.1)	131 (0.2)			

HRT – hormone replacement therapy; **NSAID** – nonsteroidal anti-inflammatory drugs; **OC** – oral contraceptives; **Pre-MP** – pre-menopausal women; **Post-MP** – post-menopausal women; **SD** – standard deviation; **n (%)** – number (percentage from total per column); **#** – tertile cut-offs for Townsend deprivation index (-3.298 and -1.067 for women; -3.295 and -0.975 for men).

Table S3 Associations of BMI with platelet parameters

			Platelet count		MPV		PDW
Group	Category	Count	$SD_{difference}$ (95% CI)	Count	$SD_{difference}$ (95% CI)	Count	$SD_{difference}$ (95% CI)
**Women**	Overall	125,435	0.080 (0.074 to 0.086)***	125,433	0.005 (-0.001 to 0.011)	125,433	0.005 (-0.001 to 0.011)
**Men**	Overall	114,760	-0.023 (-0.029 to -0.017)***	114,758	0.024 (0.018 to 0.030)***	114,758	0.053 (0.047 to 0.059)***
p_{sex}			**$3*10^{-129}$**		$2*10^{-4}$		**$1*10^{-25}$**
**Women: Pre-MP**	Overall	37,917	0.135 (0.125 to 0.146)***	37,916	-0.034 (-0.044 to -0.023)***	37,916	-0.036 (-0.047 to -0.026)***
**Women: Post-MP**	Overall	75,491	0.043 (0.036 to 0.051)***	75,490	0.030 (0.022 to 0.038)***	75,490	0.033 (0.025 to 0.041)***
$p_{women\ MP}$			**$4*10^{-61}$**		**$7*10^{-26}$**		**$3*10^{-29}$**
**Men: <52 years**	Overall	44,593	0.015 (0.006 to 0.024)**	44,592	0.014 (0.005 to 0.024)*	44,592	0.044 (0.035 to 0.053)***
**Men: ≥52 years**	Overall	70,167	-0.052 (-0.060 to -0.044)***	70,166	0.032 (0.024 to 0.040)***	70,166	0.061 (0.053 to 0.069)***
$p_{men\ age}$			**$3*10^{-36}$**		0.0003		0.004
**by Alcohol** w / m			$6*10^{-5}$ / **$1*10^{-15}$**		0.003 / 0.001		0.004 / 0.043
**Women**	≤3 times/month	39,662	0.090 (0.080 to 0.099)***	39,661	-0.004 (-0.014 to 0.005)	39,661	-0.006 (-0.015 to 0.004)
**Men**	≤3 times/month	21,648	0.003 (-0.009 to 0.016)	21,648	0.020 (0.008 to 0.033)*	21,648	0.037 (0.024 to 0.049)***
p_{sex}			**$9*10^{-28}$**		0.001		**$3*10^{-8}$**
**Women**	≤4 times/week	63,804	0.078 (0.070 to 0.087)***	63,803	0.006 (-0.002 to 0.015)	63,803	0.009 (0.000 to 0.018)*
**Men**	≤4 times/week	63,748	-0.017 (-0.025 to -0.009)**	63,746	0.016 (0.008 to 0.024)**	63,746	0.055 (0.047 to 0.064)***
p_{sex}			**$2*10^{-57}$**		0.201		**$6*10^{-13}$**
**Women**	Daily	21,969	0.054 (0.039 to 0.070)***	21,969	0.027 (0.011 to 0.043)**	21,969	0.024 (0.008 to 0.041)*
**Men**	Daily	29,364	-0.065 (-0.078 to -0.052)***	29,364	0.046 (0.033 to 0.059)***	29,364	0.065 (0.052 to 0.077)***
p_{sex}			**$7*10^{-32}$**		0.159		0.0009
**by Smoking** w / m			**$4*10^{-9}$** / **$4*10^{-13}$**		$2*10^{-5}$ / 0.0004		**$1*10^{-8}$** / 0.221
**Women**	Never	55,926	0.097 (0.088 to 0.105)***	55,925	-0.008 (-0.016 to 0.001)	55,925	-0.005 (-0.014 to 0.004)
**Men**	Never	42,770	-0.004 (-0.014 to 0.006)	42,769	0.018 (0.008 to 0.028)**	42,769	0.048 (0.038 to 0.058)***
p_{sex}			**$5*10^{-51}$**		0.004		**$8*10^{-13}$**
**Women**	Former occasional	36,310	0.082 (0.071 to 0.093)***	36,310	0.009 (-0.003 to 0.020)	36,310	-0.008 (-0.019 to 0.004)
**Men**	Former occasional	31,879	-0.007 (-0.019 to 0.005)	31,878	0.015 (0.003 to 0.027)*	31,878	0.054 (0.042 to 0.066)***
p_{sex}			**$8*10^{-33}$**		0.457		**$9*10^{-13}$**
**Women**	Former regular	22,561	0.057 (0.043 to 0.070)***	22,560	0.011 (-0.003 to 0.024)	22,560	0.028 (0.014 to 0.042)**
**Men**	Former regular	25,904	-0.044 (-0.056 to -0.031)***	25,904	0.029 (0.016 to 0.042)**	25,904	0.048 (0.035 to 0.061)***
p_{sex}			**$2*10^{-27}$**		0.025		0.017
**Women**	Current	10,638	0.039 (0.018 to 0.059)**	10,638	0.044 (0.024 to 0.065)**	10,638	0.050 (0.030 to 0.070)**
**Men**	Current	14,207	-0.070 (-0.087 to -0.053)***	14,207	0.048 (0.032 to 0.065)***	14,207	0.072 (0.056 to 0.088)***
p_{sex}			**$1*10^{-13}$**		0.953		0.211

BMI – body mass index; **CI** – confidence interval; **Count** – number of participants with available platelet measurements per category; **Post-MP** – post-menopausal; **Pre-MP** – pre-menopausal; **SD** – standard deviation.

$SD_{differences}$ (95% CI) in platelet parameters per one SD increment of BMI from multivariable linear regression models including each platelet parameter as an outcome variable (sex-specific z-scores, following log-transformation) and BMI, ABSI, and HI as exposure variables (sex-specific z-scores), with adjustment for height, age, weight change (last year), smoking status (except for subgroups), alcohol consumption (except for subgroups), physical activity, Townsend deprivation index, region of the assessment centre, time of blood collection, fasting time, use of nonsteroidal anti-inflammatory drugs, paracetamol use, menopausal status (women overall), hormonal replacement therapy use (women overall and Post-MP), oral contraceptives use and age at the last live birth (all women). Separate analyses were performed within subgroups by alcohol consumption and smoking status, individually for women and men. Plots are shown in Figure 1.

p_{sex} – p-value for the interaction term of BMI with sex, from a model including women (reference) and men, with adjustment for ABSI, HI, covariates (except female-specific), and including an interaction term between age and sex, to account for potential differences by menopausal status in women ($p<1*10^{-6}$ bold).

$p_{women\ MP\ /\ men\ age}$ – p-value for the interaction term of BMI with menopausal status, from a model including Pre-MP (reference) and Post-MP women, or of BMI with age (continuous), from a model including all men, with adjustment for ABSI, HI, and covariates ($p<1*10^{-6}$ bold).

$^{W/M}$ p-value (women / men) from likelihood ratio tests comparing models with and without an interaction term of BMI with either alcohol consumption or smoking status, separately for women and men, with adjustment for ABSI, HI, and covariates ($p<1*10^{-6}$ bold).

***** $p<0.05$; ****** $p<0.001$; ******* $p<1*10^{-6}$ p-value from Wald test for the individual term.

Table S4 Associations of ABSI and HI with platelet parameters

		**Platelet count**		**MPV**		**PDW**
Group	Count	$SD_{difference}$ (95% CI)	Count	$SD_{difference}$ (95% CI)	Count	$SD_{difference}$ (95% CI)
**ABSI**						
**Women**	125,435	0.070 (0.064 to 0.075)***	125,433	-0.036 (-0.042 to -0.031)***	125,433	0.014 (0.009 to 0.020)***
**Men**	114,760	0.059 (0.053 to 0.065)***	114,758	-0.012 (-0.018 to -0.005)**	114,758	0.007 (0.001 to 0.013)*
p_{sex}		0.001		**$1*10^{-8}$**		0.323
**Women: Pre-MP**	37,917	0.084 (0.073 to 0.095)***	37,916	-0.045 (-0.055 to -0.034)***	37,916	0.006 (-0.004 to 0.017)
**Women: Post-MP**	75,491	0.065 (0.057 to 0.072)***	75,490	-0.032 (-0.040 to -0.025)***	75,490	0.017 (0.010 to 0.024)**
$p_{women\ MP}$		**$5*10^{-7}$**		0.002		0.007
**Men: <52 years**	44,593	0.068 (0.058 to 0.078)***	44,592	-0.015 (-0.025 to -0.004)*	44,592	0.008 (-0.002 to 0.019)
**Men: ≥52 years**	70,167	0.053 (0.045 to 0.061)***	70,166	-0.010 (-0.018 to -0.002)*	70,166	0.006 (-0.002 to 0.014)
$p_{men\ age}$		**$1*10^{-7}$**		0.023		0.577
**HI**						
**Women**	125,435	-0.029 (-0.034 to -0.023)***	125,433	0.039 (0.034 to 0.045)***	125,433	-0.017 (-0.023 to -0.012)***
**Men**	114,760	-0.014 (-0.020 to -0.008)**	114,758	0.021 (0.015 to 0.027)***	114,758	-0.016 (-0.022 to -0.010)***
p_{sex}		0.0009		0.0001		0.491
**Women: Pre-MP**	37,917	-0.031 (-0.042 to -0.021)***	37,916	0.039 (0.028 to 0.049)***	37,916	-0.007 (-0.017 to 0.004)
**Women: Post-MP**	75,491	-0.027 (-0.034 to -0.020)***	75,490	0.039 (0.031 to 0.046)***	75,490	-0.024 (-0.031 to -0.017)***
$p_{women\ MP}$		0.654		0.999		0.007
**Men: <52 years**	44,593	-0.011 (-0.020 to -0.001)*	44,592	0.021 (0.011 to 0.031)**	44,592	-0.021 (-0.031 to -0.011)**
**Men: ≥52 years**	70,167	-0.014 (-0.021 to -0.006)**	70,166	0.020 (0.012 to 0.028)***	70,166	-0.013 (-0.021 to -0.006)**
$p_{men\ age}$		0.614		0.249		0.529

ABSI – a body shape index; **CI** – confidence interval; **HI** – hip index; **MPV** – mean platelet volume; **PDW** – platelet distribution width; **Post-MP** – post-menopausal women; **Pre-MP** – pre-menopausal women; **SD** – standard deviation.

$SD_{differences}$ (95% CI) in platelet parameters per one SD increment of ABSI or HI from multivariable linear regression models including each platelet parameter as an outcome variable (sex-specific z-scores, following log-transformation) and ABSI, HI, and body mass index (BMI) as exposure variables (sex-specific z-scores), with adjustment for height, age, weight change (last year), smoking status, alcohol consumption, physical activity, Townsend deprivation index, region of the assessment centre, time of blood collection, fasting time, use of nonsteroidal anti-inflammatory drugs,

paracetamol use, menopausal status (women overall), hormonal replacement therapy use (women overall and Post-MP), oral contraceptives use and age at the last live birth (all women). Plots are shown in Figure 2.

$\mathbf{p}_{\mathbf{sex}}$ – p-values for the interaction terms of ABSI and HI with sex, from a model including women (reference) and men, with adjustment for BMI, covariates (except female-specific), and including an interaction term of age with sex, to account for potential differences by menopausal status in women ($p<1*10^{-6}$ bold).

$\mathbf{p}_{\mathbf{women\ MP}}$ – p-values for the interaction terms of ABSI and HI with menopausal status, from a model including Pre-MP (reference) and Post-MP women, with adjustment for BMI and covariates ($p<1*10^{-6}$ bold).

$\mathbf{p}_{\mathbf{men\ age}}$ – p-values for the interaction terms of ABSI and HI with age (continuous), from a model including all men, with adjustment for BMI and covariates (except female-specific) ($p<1*10^{-6}$ bold).

* $p<0.05$; ** $p<0.001$; *** $p<1*10^{-6}$ p-value from Wald test for the individual term.

Table S5 Associations of body shape phenotypes with platelet parameters

Group	Phenotype	Platelet count		MPV		PDW	
		Count	$SD_{difference}$ (95% CI)	Count	$SD_{difference}$ (95% CI)	Count	$SD_{difference}$ (95% CI)
**ABSI-by-HI overall** $^{p\ sex\ shape}$			**$3*10^{-8}$**		**$4*10^{-8}$**		0.016
**Women** [A]	Pear	33,369	reference	33,369	reference	33,369	reference
	Slim	28,836	0.045 (0.029 to 0.060)***	28,836	-0.065 (-0.081 to -0.049)***	28,836	0.015 (-0.001 to 0.031)
	Wide	36,548	0.122 (0.107 to 0.137)***	36,546	-0.056 (-0.071 to -0.041)***	36,546	0.015 (0.000 to 0.029)
	Apple	26,682	0.160 (0.144 to 0.176)***	26,682	-0.118 (-0.134 to -0.102)***	26,682	0.050 (0.034 to 0.066)***
p_{shape}			**$4*10-^{104}$**		**$2*10^{-44}$**		**$3*10^{-8}$**
**Men** [A]	Pear	30,990	reference	30,990	reference	30,990	reference
	Slim	35,393	0.006 (-0.009 to 0.021)	35,392	-0.024 (-0.039 to -0.008)*	35,392	0.026 (0.011 to 0.042)**
	Wide	30,391	0.075 (0.059 to 0.091)***	30,390	-0.007 (-0.023 to 0.009)	30,390	0.012 (-0.004 to 0.028)
	Apple	17,986	0.096 (0.078 to 0.115)***	17,986	-0.043 (-0.062 to -0.024)**	17,986	0.014 (-0.004 to 0.033)
p_{shape}			**$2*10^{-37}$**		$2*10^{-5}$		0.010
**BMI-by-ABSI-by-HI** $^{p\ sex\ shape\text{-}by\text{-}size}$			**$2*10^{-103}$**		**$8*10^{-10}$**		**$5*10^{-29}$**
**Women: NW** [B]	Pear	17,377	reference	17,377	reference	17,377	reference
	Slim	14,315	0.059 (0.037 to 0.081)***	14,315	-0.078 (-0.100 to -0.056)***	14,315	0.003 (-0.019 to 0.026)
	Wide	15,966	0.145 (0.124 to 0.167)***	15,966	-0.063 (-0.084 to -0.041)***	15,966	-0.004 (-0.026 to 0.017)
	Apple	9,863	0.203 (0.178 to 0.227)***	9,863	-0.121 (-0.146 to -0.096)***	9,863	0.013 (-0.011 to 0.038)
**Women: OW** [B]	Pear	10,952	0.123 (0.099 to 0.147)***	10,952	0.010 (-0.014 to 0.034)	10,952	-0.012 (-0.036 to 0.012)
	Slim	10,448	0.160 (0.136 to 0.185)***	10,448	-0.048 (-0.073 to -0.024)**	10,448	-0.001 (-0.026 to 0.023)
	Wide	12,898	0.248 (0.225 to 0.271)***	12,896	-0.055 (-0.078 to -0.032)**	12,896	-0.002 (-0.025 to 0.021)
	Apple	11,547	0.287 (0.263 to 0.310)***	11,547	-0.110 (-0.134 to -0.086)***	11,547	0.051 (0.027 to 0.075)**
**Women: OB** [B]	Pear	5,040	0.269 (0.238 to 0.301)***	5,040	-0.021 (-0.053 to 0.011)	5,040	-0.050 (-0.082 to -0.019)*
	Slim	4,073	0.283 (0.249 to 0.317)***	4,073	-0.057 (-0.091 to -0.022)*	4,073	0.013 (-0.022 to 0.047)
	Wide	7,684	0.316 (0.289 to 0.343)***	7,684	-0.040 (-0.068 to -0.013)*	7,684	0.030 (0.002 to 0.057)*
	Apple	5,272	0.316 (0.286 to 0.347)***	5,272	-0.121 (-0.152 to -0.090)***	5,272	0.062 (0.030 to 0.093)**
$p_{shape\text{-}by\text{-}size}$			**$1*10^{-9}$**		0.286		$2*10^{-4}$
**Men: NW** [B]	Pear	10,604	reference	10,604	reference	10,604	reference
	Slim	9,653	0.004 (-0.023 to 0.031)	9,652	-0.002 (-0.030 to 0.025)	9,652	0.004 (-0.024 to 0.031)
	Wide	9,717	0.090 (0.062 to 0.117)***	9,717	-0.027 (-0.055 to 0.000)	9,717	0.000 (-0.028 to 0.027)
	Apple	4,352	0.138 (0.104 to 0.173)***	4,352	-0.059 (-0.094 to -0.023)*	4,352	-0.015 (-0.050 to 0.020)
**Men: OW** [B]	Pear	15,116	0.002 (-0.023 to 0.026)	15,116	0.021 (-0.004 to 0.046)	15,116	0.063 (0.038 to 0.087)***
	Slim	19,029	0.007 (-0.016 to 0.031)	19,029	-0.006 (-0.030 to 0.018)	19,029	0.104 (0.080 to 0.128)***
	Wide	14,728	0.062 (0.038 to 0.087)***	14,727	0.026 (0.001 to 0.052)*	14,727	0.092 (0.067 to 0.117)***
	Apple	9,673	0.098 (0.071 to 0.126)***	9,673	-0.017 (-0.045 to 0.011)	9,673	0.090 (0.063 to 0.118)***
**Men: OB** [B]	Pear	5,270	-0.048 (-0.081 to -0.015)*	5,270	0.066 (0.033 to 0.099)**	5,270	0.139 (0.105 to 0.172)***
	Slim	6,711	-0.040 (-0.071 to -0.010)*	6,711	0.021 (-0.010 to 0.052)	6,711	0.162 (0.131 to 0.192)***
	Wide	5,946	0.037 (0.005 to 0.069)*	5,946	0.060 (0.028 to 0.092)**	5,946	0.128 (0.095 to 0.160)***
	Apple	3,961	-0.004 (-0.041 to 0.032)	3,961	0.030 (-0.007 to 0.066)	3,961	0.160 (0.123 to 0.196)***
$p_{shape\text{-}by\text{-}size}$			0.002		0.027		0.158

		Platelet count		MPV		PDW	
Group	Phenotype	Count	$SD_{difference}$ (95% CI)	Count	$SD_{difference}$ (95% CI)	Count	$SD_{difference}$ (95% CI)
**Women: NW** [B]	Apple vs Pear		0.203 (0.178 to 0.227)***		-0.121 (-0.146 to -0.096)***		0.013 (-0.011 to 0.038)
**Women: OW** [B]	Apple vs Pear		0.164 (0.138 to 0.190)***		-0.120 (-0.146 to -0.094)***		0.063 (0.037 to 0.089)**
**Women: OB** [B]	Apple vs Pear		0.047 (0.009 to 0.085)*		-0.100 (-0.138 to -0.061)***		0.112 (0.073 to 0.150)***
**Men: NW** [B]	Apple vs Pear		0.138 (0.104 to 0.173)***		-0.059 (-0.094 to -0.023)*		-0.015 (-0.050 to 0.020)
**Men: OW** [B]	Apple vs Pear		0.096 (0.071 to 0.122)***		-0.038 (-0.064 to -0.012)*		0.028 (0.002 to 0.053)*
**Men: OB** [B]	Apple vs Pear		0.043 (0.003 to 0.084)*		-0.036 (-0.078 to 0.005)		0.021 (-0.020 to 0.062)

ABSI – a body shape index (cutoffs ≥73 for women; ≥80 for men); **Apple** – large-ABSI-small-HI; **BMI** – body mass index; **CI** – confidence interval; **HI** – hip index (cutoffs ≥64 for women; ≥49 for men); **MPV** – mean platelet volume; **NW** – normal weight (BMI≥18.5 to BMI<25 kg/m^2); **OB** – obese (BMI≥30 to BMI<45 kg/m^2); **OW** – overweight (BMI≥25 to BMI<30 kg/m^2); **PDW** – platelet distribution width; **Pear** – small-ABSI-large-HI; **SD** – standard deviation; **Slim** – small-ABSI-small-HI; **Wide** – large-ABSI-large-HI.

$SD_{differences}$ (95% CI) in platelet parameters compared to the reference category from multivariable linear regression models including each platelet parameter as an outcome variable (sex-specific z-scores, following log-transformation) and as exposures, ABSI-by-HI ("pear" reference) and BMI categories (model A), or BMI-by-ABSI-by-HI ("pear" NW reference) (model B), with adjustment for height, age, weight change (last year), smoking status, alcohol consumption, physical activity, Townsend deprivation index, region of the assessment centre, time of blood collection, fasting time, use of nonsteroidal anti-inflammatory drugs, paracetamol use, and for women, menopausal status, hormonal replacement therapy use, oral contraceptives use, and age at the last live birth. Plots are shown in Figure 3.

$p_{shape / shape\text{-}by\text{-}size}$ – p-values for the association with body shape overall ($_{shape}$) or for heterogeneity of the associations with body shape according to BMI category ($_{shape\text{-}by\text{-}size}$), from likelihood ratio tests (separately for women and men), comparing fully adjusted models including BMI categories with and without ABSI-by-HI ($_{shape}$), or the fully adjusted additive model including ABSI-by-HI and BMI categories, with the interaction model including BMI-by-ABSI-by-HI ($_{shape\text{-}by\text{-}size}$) ($p<1*10^{-6}$ bold).

$p_{sex\ shape / sex\ shape\text{-}by\text{-}size}$ – p-values evaluating men vs women, from likelihood ratio tests comparing models including women (reference) and men with and without an interaction term of ABSI-by-HI ($_{sex\ shape}$) or BMI-by-ABSI-by-HI ($_{sex\ shape\text{-}by\text{-}size}$) with sex, adjusted for covariates (except female-specific), and including an interaction term of age with sex ($p<1*10^{-6}$ bold).

* $p<0.05$; ** $p<0.001$; *** $p<1*10^{-6}$ p-value from Wald test for the individual term.

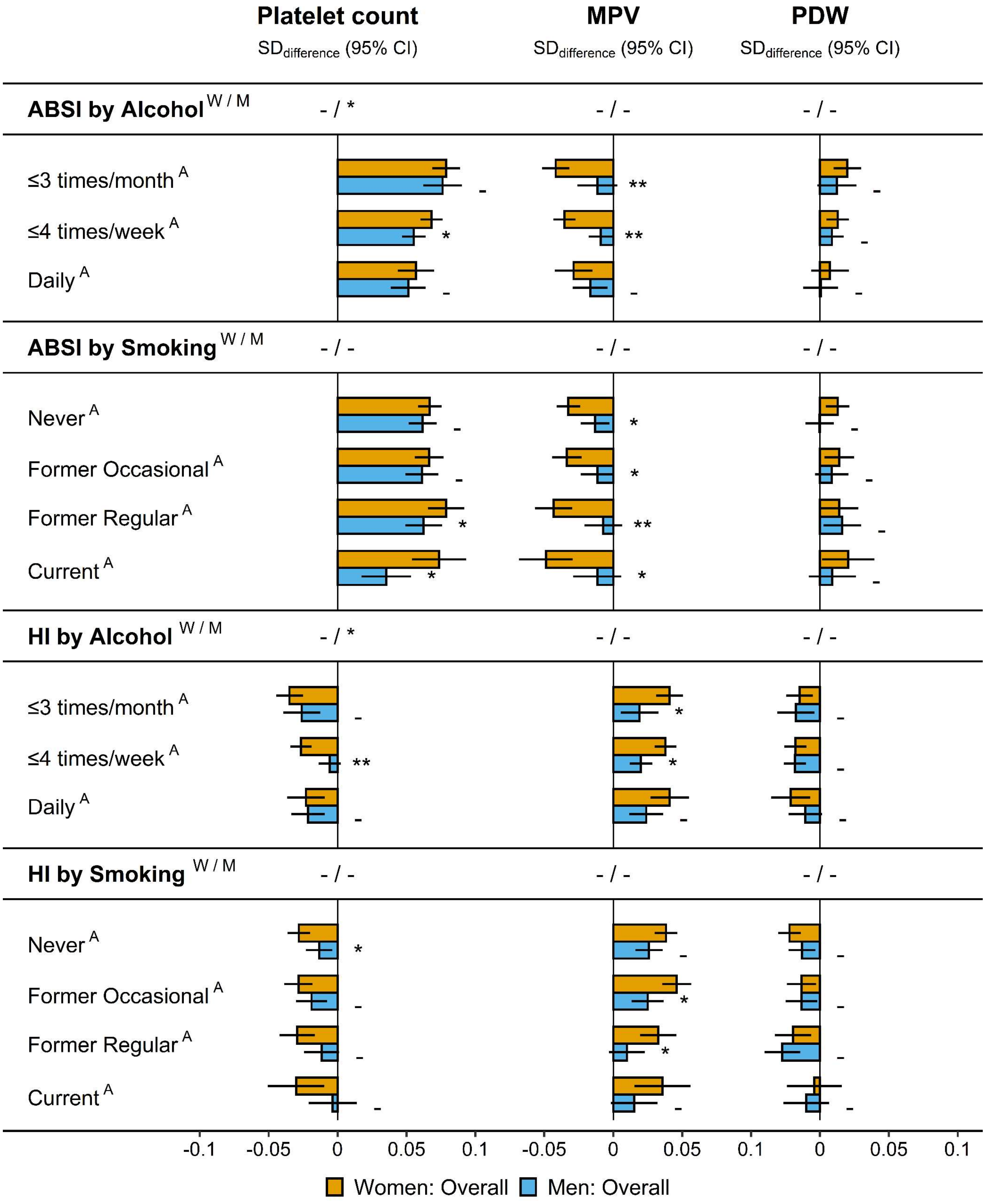

Fig. S1 Associations of ABSI and HI with platelet parameters (subgroups by alcohol consumption and smoking status)

ABSI – a body shape index; **BMI** – body mass index; **CI** – confidence interval; **HI** – hip index; **MPV** – mean platelet volume; **PDW** – platelet distribution width; **SD** – standard deviation.

$SD_{differences}$ (95% CI) in platelet parameters per one SD increment of ABSI or HI from multivariable linear regression models including each platelet parameter as an outcome variable (sex-specific z-

scores, following log-transformation) and BMI, ABSI, and HI as exposure variables (sex-specific z-scores), with adjustment for height, age, weight change (last year), smoking status (for models by alcohol consumption), alcohol consumption (for models by smoking status), physical activity, Townsend deprivation index, region of the assessment centre, time of blood collection, fasting time, use of nonsteroidal anti-inflammatory drugs, paracetamol use, and for women, menopausal status, hormonal replacement therapy use, oral contraceptives use, and age at the last live birth. Separate analyses were performed within subgroups by alcohol consumption and smoking status, individually for women and men.

[A] p-value for the interaction terms of ABSI and HI with sex, from a model including women (reference) and men, with adjustment for BMI and covariates (except female-specific), and including an interaction term of age with sex, to account for potential differences by menopausal status in women.

[W / M] p-value (women / men) derived from likelihood ratio tests comparing models with and without interaction terms of either ABSI or HI with either alcohol consumption or smoking status, separately for women and men, with adjustment for BMI and covariates.

p-values - $p \geq 0.05$; ***** $p<0.05$; ****** $p<0.001$; ******* $p<1*10^{-6}$

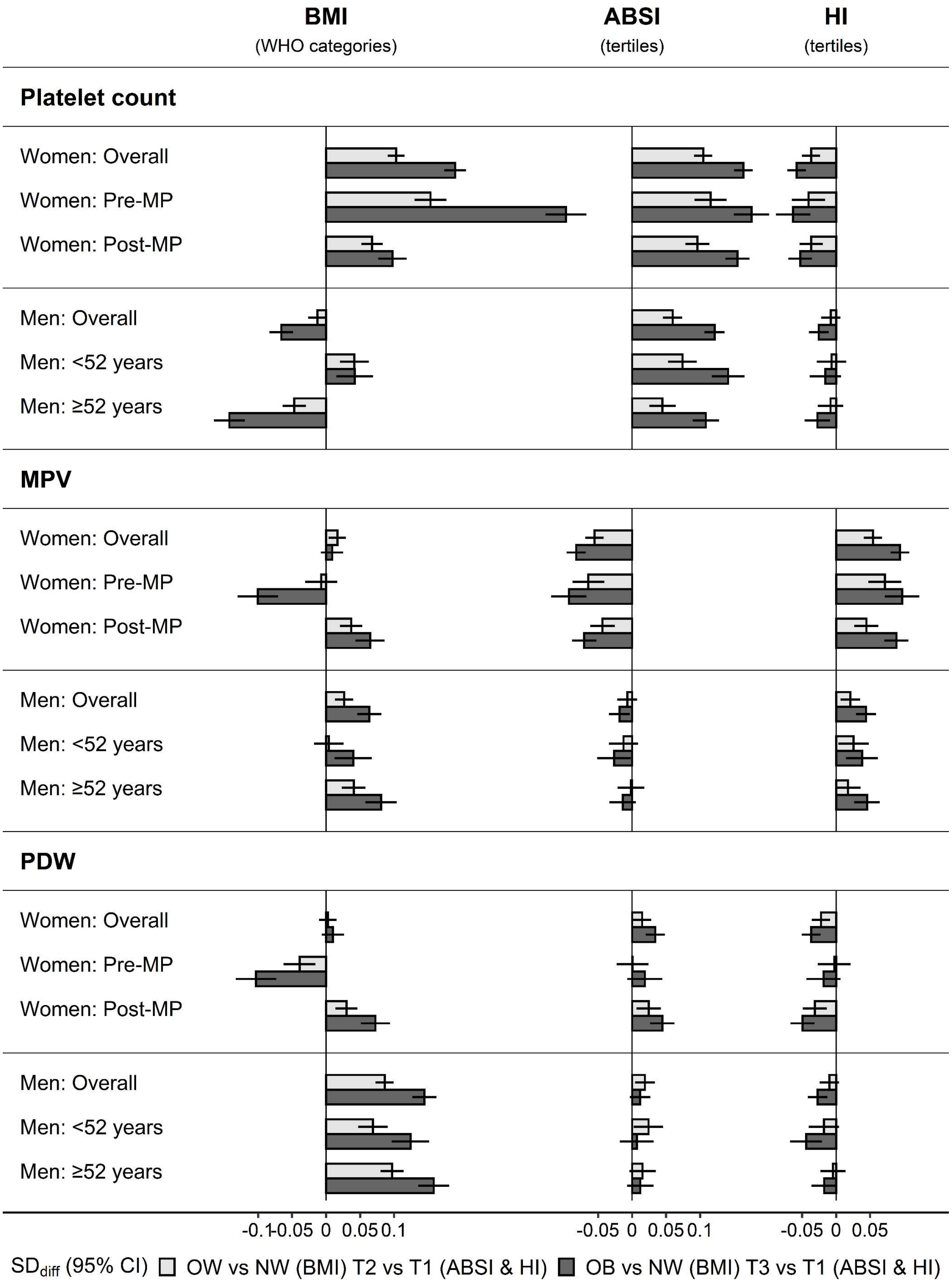

Fig. S2 Associations of anthropometric index categories with platelets

ABSI – a body shape index; **BMI** – body mass index; **CI** – confidence interval; **HI** – hip index; **MPV** – mean platelet volume; **NW** – normal weight (BMI≥18.5 to BMI<25 kg/m^2); **OB** – obese (BMI≥30 to BMI<45 kg/m^2); **OW** – overweight (BMI≥25 to BMI<30 kg/m^2); **PDW** – platelet distribution width;

Post-MP – post-menopausal women; **Pre-MP** – pre-menopausal women; **SD** – standard deviation; **T1-T3** – tertiles (cutoffs for ABSI: 71.088 and 75.133 for women; 77.513 and 80.920 for men; cutoffs for HI: 63.362 and 65.261 for women; 48.490 and 49.759 for men).

$SD_{differences}$ (95% CI) in platelet parameters compared to the reference category obtained from multivariable linear regression models including each platelet parameter as an outcome variable (sex-specific z-scores, following log-transformation) and as exposures, categories of BMI (NW reference) and tertiles of ABSI and HI (T1 reference), with adjustment for height, age, weight change (last year), smoking status, alcohol consumption, physical activity, Townsend deprivation index, region of the assessment centre, time of blood collection, fasting time, use of nonsteroidal anti-inflammatory drugs, paracetamol use, menopausal status (women overall), hormonal replacement therapy use (women overall and Post-MP), oral contraceptives use and age at the last live birth (all women).

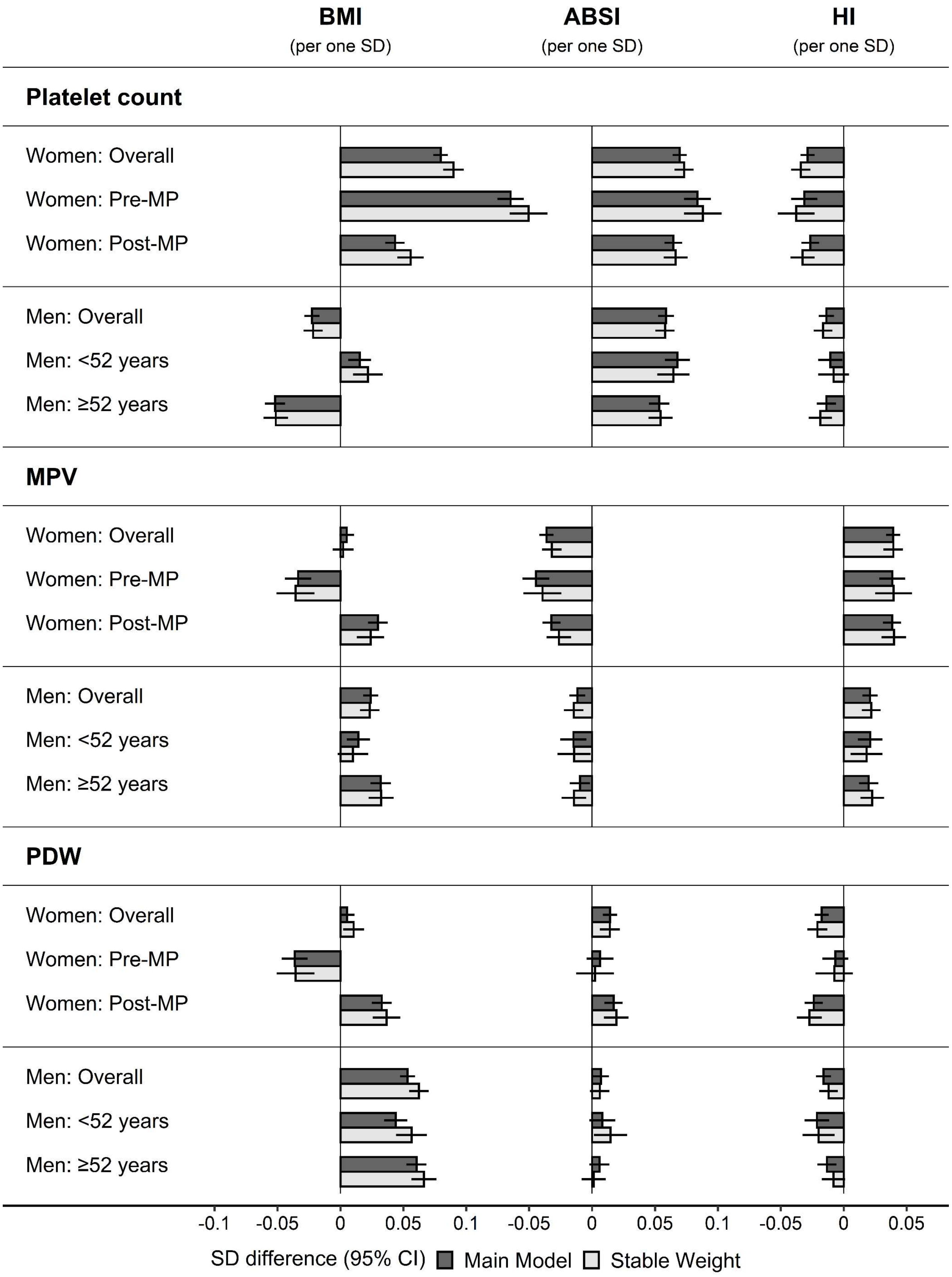

Fig. S3 Associations of anthropometric indices with platelets in participants with stable weight

ABSI – a body shape index; **BMI** – body mass index; **CI** – confidence interval; **HI** – hip index; **MPV** – mean platelet volume; **PDW** – platelet distribution width; **Post-MP** – post-menopausal women; **Pre-MP** – pre-menopausal women; **SD** – standard deviation.

SD difference (95% CI) in platelet parameters per one SD increment of BMI, ABSI, or HI from multivariable linear regression models including each platelet parameter as an outcome variable (sex-specific z-scores, following log-transformation) and BMI, ABSI, and HI as exposure variables (sex-specific z-scores), with adjustment for height, age, weight change within the last year preceding recruitment (main analysis only), smoking status, alcohol consumption, physical activity, Townsend deprivation index, region of the assessment centre, time of blood collection, fasting time, use of nonsteroidal anti-inflammatory drugs, paracetamol use, menopausal status (women overall), hormonal replacement therapy use (women overall and Post-MP), oral contraceptives use and age at the last live birth (all women).

The plots show a sensitivity analysis, restricted to participants with self-reported stable weight within the last year preceding recruitment, in comparison to the main analysis.

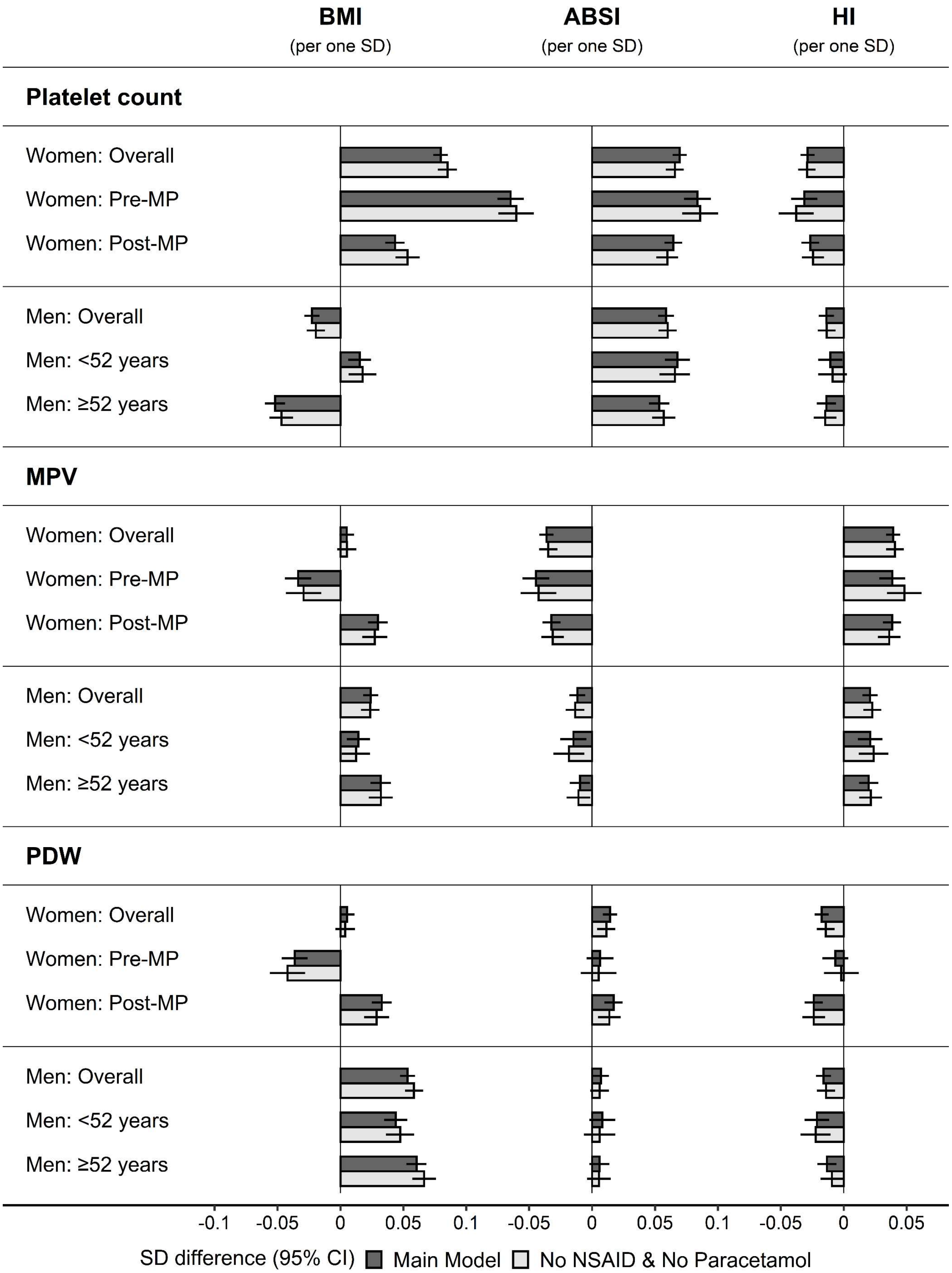

Fig. S4 Associations of anthropometric indices with platelets in participants without NSAID or paracetamol use

ABSI – a body shape index; **BMI** – body mass index; **CI** – confidence interval; **HI** – hip index; **MPV** – mean platelet volume; **NSAID** – non-steroidal anti-inflammatory drugs; **PDW** – platelet

distribution width; **Post-MP** – post-menopausal women; **Pre-MP** – pre-menopausal women; **SD** – standard deviation.

SD difference (95% CI) in platelet parameters per one SD increment of BMI, ABSI, or HI from multivariable linear regression models including each platelet parameter as an outcome variable (sex-specific z-scores, following log-transformation) and BMI, ABSI, and HI as exposure variables (sex-specific z-scores), with adjustment for height, age at recruitment, weight change (last year), smoking status, alcohol consumption, physical activity, Townsend deprivation index, region of the assessment centre, time of blood collection, fasting time, use of nonsteroidal anti-inflammatory drugs and paracetamol (main analysis only), menopausal status (women overall), hormonal replacement therapy use (women overall and Post-MP), oral contraceptives use and age at the last live birth (all women).

The plots show a sensitivity analysis, restricted to participants receiving no NSAIDs and no paracetamol, in comparison to the main analysis.

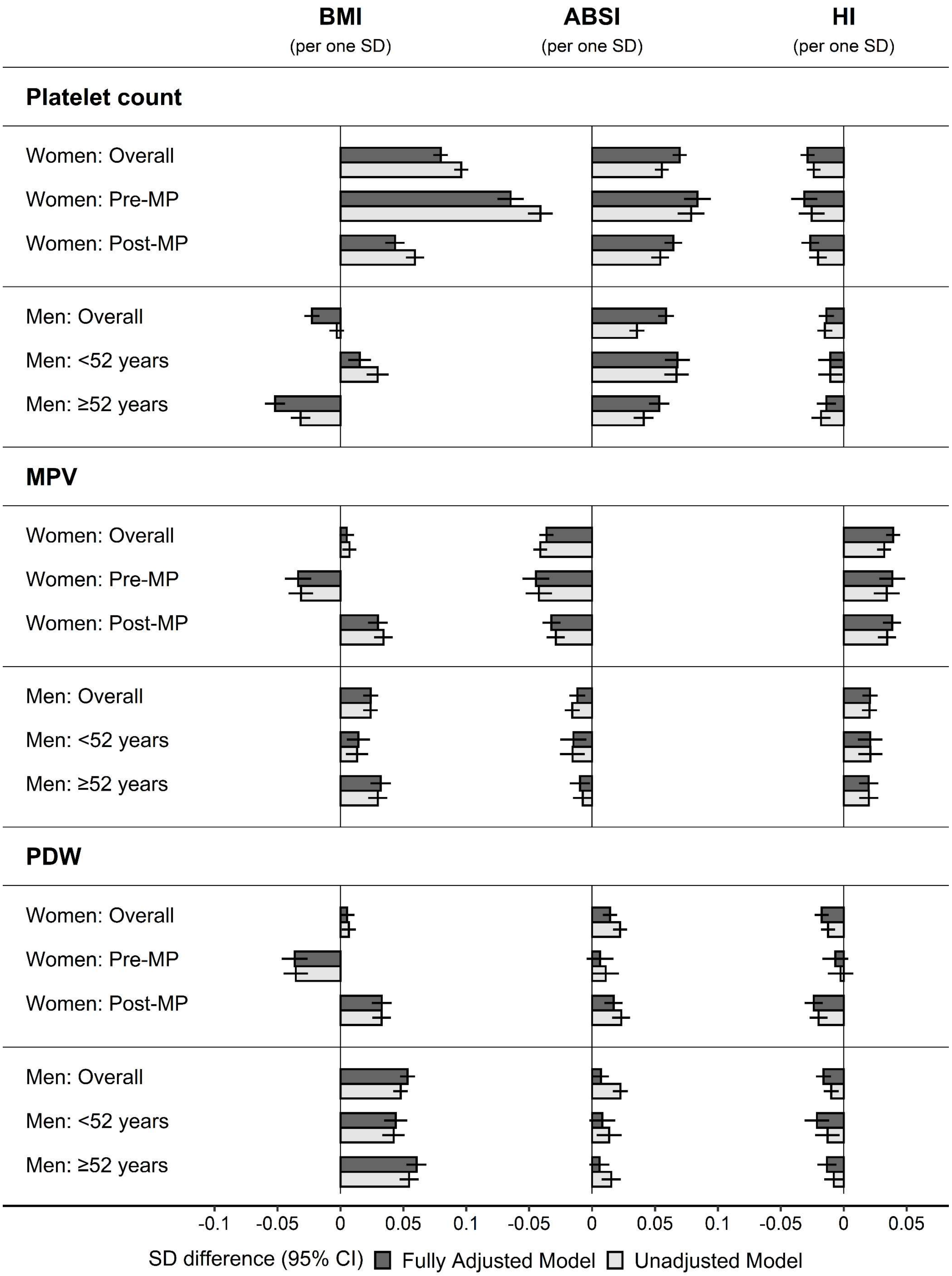

Fig. S5 Unadjusted associations of anthropometric indices with platelets

ABSI – a body shape index; **BMI** – body mass index; **CI** – confidence interval; **HI** – hip index; **MPV** – mean platelet volume; **PDW** – platelet distribution width; **Post-MP** – post-menopausal women; **Pre-MP** – pre-menopausal women; **SD** – standard deviation.

SD difference (95% CI) in platelet parameters per one SD increment of BMI, ABSI, or HI from multivariable linear regression models including each platelet parameter as an outcome variable (sex-specific z-scores, following log-transformation) and BMI, ABSI, and HI as exposure variables (sex-specific z-scores). **Unadjusted** models included no adjustment variables. **Fully Adjusted** models, shown for comparison, represent the main analyses with adjustment for height, age, weight change (last year), smoking status, alcohol consumption, physical activity, Townsend deprivation index, region of the assessment centre, time of blood collection, fasting time, use of nonsteroidal anti-inflammatory drugs, paracetamol use, menopausal status (women overall), hormonal replacement therapy use (women overall and Post-MP), oral contraceptives use and age at the last live birth (all women).

References

The number of the cited reference correspond to the main document.

12. Christakoudi, S., Riboli, E., Evangelou, E. & Tsilidis, K. K. Associations of body shape index (ABSI) and hip index with liver, metabolic, and inflammatory biomarkers in the UK Biobank cohort. *Sci Rep.* 2022;12(1):8812. doi:10.1038/s41598-022-12284-4.

23. Christakoudi, S., Tsilidis, K. K., Evangelou, E. & Riboli, E. A Body Shape Index (ABSI), hip index, and risk of cancer in the UK Biobank cohort. *Cancer Med.* 2021;10(16):5614-28. doi:10.1002/cam4.4097.
